# Routing Protocols for Low Power and Lossy Networks in Internet of Things Applications

**DOI:** 10.3390/s19092144

**Published:** 2019-05-09

**Authors:** José V. V. Sobral, Joel J. P. C. Rodrigues, Ricardo A. L. Rabêlo, Jalal Al-Muhtadi, Valery Korotaev

**Affiliations:** 1 Instituto de Telecomunicações, Universidade da Beira Interior, 6201-001 Covilhã, Portugal; jose.sobral@it.ubi.pt; 2 Federal Institute of Maranhão (IFMA), São Luís-MA 65010-030, Brazil; 3 National Institute of Telecommunications (Inatel), Santa Rita do Sapucaí-MG 37540-000, Brazil; 4 Federal University of Piauí, Teresina-PI 64049-550, Brazil; ricardoalr@ufpi.edu.br; 5 College of Computer and Information Sciences (CCIS), King Saud University, Riyadh 12372, Saudi Arabia; jalal@ccis.edu.sa; 6 ITMO University, St. Petersburg 197101, Russia; korotaev@grv.ifmo.ru

**Keywords:** Internet of Things, low-power and lossy network, LOADng, routing protocol, RPL

## Abstract

The emergence of the Internet of Things (IoT) and its applications has taken the attention of several researchers. In an effort to provide interoperability and IPv6 support for the IoT devices, the Internet Engineering Task Force (IETF) proposed the 6LoWPAN stack. However, the particularities and hardware limitations of networks associated with IoT devices lead to several challenges, mainly for routing protocols. On its stack proposal, IETF standardizes the RPL (IPv6 Routing Protocol for Low-Power and Lossy Networks) as the routing protocol for Low-power and Lossy Networks (LLNs). RPL is a tree-based proactive routing protocol that creates acyclic graphs among the nodes to allow data exchange. Although widely considered and used by current applications, different recent studies have shown its limitations and drawbacks. Among these, it is possible to highlight the weak support of mobility and P2P traffic, restrictions for multicast transmissions, and lousy adaption for dynamic throughput. Motivated by the presented issues, several new solutions have emerged during recent years. The approaches range from the consideration of different routing metrics to an entirely new solution inspired by other routing protocols. In this context, this work aims to present an extensive survey study about routing solutions for IoT/LLN, not limited to RPL enhancements. In the course of the paper, the routing requirements of LLNs, the initial protocols, and the most recent approaches are presented. The IoT routing enhancements are divided according to its main objectives and then studied individually to point out its most important strengths and weaknesses. Furthermore, as the main contribution, this study presents a comprehensive discussion about the considered approaches, identifying the still remaining open issues and suggesting future directions to be recognized by new proposals.

## 1. Introduction

In the near future, it is expected that all things will be able communicate among themselves through the Internet. It is easy to perceive this evolution in a time when things are more and more connected to the global computer network. This reason leads to the emergence of a new paradigm called the Internet of Things (IoT) [1]. According to [2], the core idea of the IoT is the pervasive presence of a variety of objects and things around us that can exchange information to reach a common objective. According to [3], in 2025, Internet nodes will be present in people’s daily routines in the form of furniture, papers, televisions, refrigerators, and food packaging.

According to [4], IoT is a multidisciplinary domain that encompasses a vast number of topics, including purely technical challenges and a mix of technical and social tasks, as well as social and enterprise efforts. Ambient and personal care monitoring, industrial planning monitoring, including agriculture, smart environments, and smart cities are examples of IoT applications [5,6,7].

Wireless Sensor Networks (WSN) are an essential component of the IoT because they enable the development of applications with a capacity to sense several variables related to the environment in which the devices are inserted [8]. WSN is a specific kind of LoWPAN (Low-power Wireless Personal Area Network) composed by nodes equipped with different types of sensors (e.g., temperature, humidity, and luminosity). To begin with, the interoperability between WSN and the Internet was limited due to the absence of IP communication infrastructure. Thus, seeking to solve this problem, several works were dedicated to proposing a structure that could provide the use of IP over LoWPAN [9].

Aiming to provide a standard solution for this problem, in October 2004, IETF (Internet Engineering Task Force) proposed a Working Group (WG) named 6LoWPAN (IPv6 over Low-power Wireless Personal Area Networks). The 6LoWPAN working group’s objective was to create ways to enable the use of IPv6 over IEEE 802.15.4 networks. Thus, 6LoWPAN suggested the inclusion of an adaption layer in the IP stack between the network and data link layer. This new adaption layer permits the fragmentation and defragmentation of IPv6 packets in IEEE 802.15.4 frames, realizing the compression of the IPv6 head [10]. After the publication of six RFC (Request For Comments) documents (RFC 4919 [10], RFC 4944 [11], RFC 6282 [12], RFC 6568 [13], RFC 6606 [14], RFC 6775 [15]), the IETF defined the end of the 6LoWPAN working group as January 2014. The contributions of 6LoWPAN WG allowed the use of an IP-based network of tiny devices and, consequently, the existence of IoT applications [16]. Through the work of 6LoWPAN WG, a new working group was created to study routing solutions for the emerging Low-power and Lossy Networks (LLN) [17].

Created in February 2008, the Routing over Low-power and Lossy networks (RoLL) working group initially defined the routing requirements for urban LLNs [18], industrial LLNs [19], home automation LLNs [20], and building automation LLNs [21]. In parallel to the definition of the routing requirements, the RoLL working group initiated a study to verify whether the IETF standard routing protocols could supply the identified requirements [22]. Through the first conclusions, the RoLL detected that the existent routing solutions could not satisfy the requirements of LLNs. Since then, the working group has studied new routing solutions to provide the specific requirements of several types of LLN applications [17,23]. In March 2012, the RoLL working group defined the IPv6 Routing Protocol for LLNs (RPL) as the standard routing protocol for LLN. Presented in the RFC 6550 [24], RPL is a proactive tree-based routing protocol that builds a directed acyclic graph between the leaf nodes and the border route (sink node). Although considered as the standard routing protocol for IoT networks [25], since its creation, RPL has presented several drawbacks, and new solutions have been emerging to solve them.

The routing protocols for low power networks are summarized in several survey works. The works in [26,27,28] presented surveys about initial routing protocols for 6LoWPAN. A study about a secure routing protocol for IoT was presented in [29]. Considering the Smart Grid (SG) as a kind of IoT application inside the smart city domain [30,31], the authors in [32,33] showed survey studies about the routing protocols and network challenges for SG. Most recently, [34,35,36] have committed to surveying the existent routing solutions, considering improvements purely for RPL.

The weaknesses of the current solutions have motivated the emergence of an increasing number of new protocols and adaptations that seek to improve the network performance and avoid faults. Further, with the RPL-based improvements, it is also possible to find routing solutions for IoT networks based on other routing protocols, e.g., the AODV (Ad-hoc On-demand Distance Vector) routing protocol [37]. Thus, this work aims to present an extensive survey about the existent routing solutions for low-power networks that can be used in IoT applications. Different from the current surveys existent in the literature, this work does not only consider RPL-based approaches, but all relevant solutions introduced during recent years. Thus, the main contributions of this work are summarized as follows:

- Presents the routing requirements identified by the IETF for LLN applications.
- Overviews the initial routing protocols considered to be adopted for LLNs.
- Identifies the issues most studied by the current proposals for routing in IoT scenarios.
- Presents a comprehensive survey showing the approach and functioning of the most relevant routing solutions for IoT/LLNs.
- Describes the studied solutions in summarizing tables and discusses the strengths and weakness of current routing solutions.
- Indicates relevant open issues and future directions to be appreciated by new routing approaches for IoT networks.

The rest of this work is organized as follows. Section 2 presents the routing requirements for applications over low-power networks. Section 3 elaborates the most-studied routing protocols for low-power and lossy networks, and Section 4 describes the most relevant routing solutions available in the literature to fulfill the requirements of IoT/LLN applications. A comprehensive discussion about the studied approaches, lessons learned, and the open issues and guidelines to be considered during the design of new solutions are addressed in Section 5. Section 6 concludes the paper.

## 2. Routing Requirements of Low-Power Networks and IoT Applications

The routing requirements of IoT/LLN networks may vary according to the specificities of applications. Thus, the RoLL working group initially presented four informational RFCs describing the necessities for different groups of LLN. In [18] were presented the routing requirements for Urban LLNs (U-LLN). This kind of LLN provides the network structure for applications executed in urban environments such as smart-metering, pollution and meteorological monitoring, and general purposes for smart cities. The document describes that U-LLNs can present three different types of devices: sensors, actuators, and routers. The actuator and sensor nodes have the capacity of performing actions and measure physical data in the environment in which they are deployed, respectively. On the other hand, routers have the function of prolonging the network’s lifetime, balancing energy consumption, building the sensing infrastructure, and providing links to the Internet.

The number of sensor and actuator nodes is, generally, higher than the number of routers. It is expected that the number of sensors is between $10^{2}$ and $10^{7}$, deployed randomly or, e.g., along a road or river. The nodes have strong hardware constraints and a limited energy source. The communication distance between two nodes can vary from a few meters to one kilometer. It is common that the communication between a sensor/actuator and a border router needs to be performed through several hops, requiring multihop routing protocols. The communication reliability between the network nodes can be affected by different aspects, such as wireless channel effects, collision in the MAC layer, and interference [18].

The RoLL presented the routing requirements for Industrial LLNs in [19]. Security, monitoring, and control in industrial environments are some examples of applications supported by this group of LLNs. Thus, wireless devices should provide easy installation and maintenance, low power, and high reliability. Existing industrial LLN applications have, generally, networks with 10–200 nodes. The packet-sending rate demand ranges from 1/s to 1/h, with an average of 1/min. It is expected that critical alarm packets can be delivered with lower latency than normal messages. Hence, the routing protocol must support different routing metrics, including link quality and throughput. Further, industrial LLN applications can require the sending of messages for specific groups of nodes or more than one base station. In this case, multicast forwarding support becomes a critical routing requirement for these networks. The capacity of support in the addition of new nodes, real-time adaption to link failures and route recomputing, and mobility support (with speeds up to 35 km/h) are other routing requirements for industrial LLNs.

Through RFC 5826 [20], the RoLL working group has defined the routing requirements for Home Automation LNNs (HA-LLN). Home automation scenarios encompass a great set of applications such as energy conservation and consumption optimization, window shade control, and healthcare [38]. Although the HA applications commonly use wired networks and power-line communication, the use of wireless networks can facilitate application expansion and possible upgrades. The routing requirements for HA-LLNs applications may vary according to the use case. For example, based on the above-mentioned informational RFC, a healthcare HA-LLN application requires a constraint-based routing, with mobility support and low convergence time. On the other hand, an alarm system HA-LLN application requires a network able to provide high scalability and convergence time. Further, the reliable support for different traffic patterns represents an indispensable feature for routing protocols in residential applications. Summarizing, a routing protocol able to support all HA-LLN applications must be ready to provide constraint-based routing and support to mobility, scalability, convergence time, manageability, and stability.

In [21], the routing requirements for Building Automation LLNs (BA-LLNs) were described. BA applications include the monitoring of several aspects such as fire alarms, elevator systems, control access, and intrusion detection systems [39]. In this context, wireless devices can increase the flexibility and reduce the deployment costs of applications. The network structure for BA applications should support at least two thousand nodes, including sensors, actuators, controllers, and user interface devices. A complete Building Management Systems (BMS) can be composed from different, smaller applications executed in several subnetworks that are connected through a backbone. Both the traffic pattern and the device density, as well as several other network peculiarities can diverge for each subnetwork. Therefore, the routing protocols must provide a self-organizing structure with zero-configuration to make the installation and replacement of devices accessible. Further, it must also provide scalability and support resource-constrained devices, mobility, addressing, manageability, dynamic route selection, and security.

During the definition of the requirements for the different LLN types, IETF studied its existent standard routing protocols. The objective was to identify whether they could attend to the routing requirements of low-power networks [22]. This study has considered the routing protocols for wired and wireless networks. Among these are OSPF (Open Shortest Path First) Version 2 [40], RIP (Routing Information Protocol) Version 2 [41], AODV [37], DYMO (Dynamic Mobile ad hoc network On-demand routing) [42], and DSR (Dynamic Source Routing) [43]. It was established that different basic criteria were derived from common requirements of LLNs applications. Thus, if at least one of the criteria was not met, the routing protocol was considered as not sufficient.

Based on the conclusions of the IETF study and with the necessity of solving the routing problems existent in LLNs, several extensions and new approaches have emerged. The next section presents the most relevant ones. This study has the objective of presenting a survey of routing solutions that may be employed in networks for IoT/LLN applications. Thus, only routing protocols for wireless networks with support for IPv6 are considered.

## 3. Routing Protocols for Low-Power and Lossy Networks

### 3.1. Initial Approaches

Following the presentation of the 6LoWPAN problem statement and goals through RFC 4919 [10], several routing protocols were proposed. The initial intention of the solutions was to offer routing solutions to provide multihop communication among IEEE 802.15.4 devices with support to IPv6. The main protocols, which are published, mainly, by means of Internet-Drafts (I-D), are briefly introduced in the following paragraphs.

The Hierarchical routing for 6LoWPAN (Hi-Low) is a hierarchical-based routing protocol that proposes the use of the dynamic address assignment scheme [44]. The basic idea of Hi-Low is to create a routing tree in which each node has a unique short address with 16 bits. In the Hi-Low topology, the network nodes are divided into three types: coordinator, router (parent), or end device (child). Each node of the network maintains a table with information about its neighbors such as its short address, device type, depth, and others. In the Hi-Low operation, a node initially seeks and tries to join an already existent network. If it finds a node that is already in the network, the node that is already part of the network should define a short address to the new node. If the network is not found, the node assumes the function of coordinator, creates a new network, and defines its short address as zero. A parent (or coordinator) node must define the address of its children nodes using Equation (1), in which MC is a parameter that defines the maximum number of children that a node can have and addressParent is the address of the parent. Hi-Low nodes frequently send beacon messages to update the neighboring information in the table. In the routing operation, a Hi-Low node uses the destination address to calculate whether the message should be sent to its parent or its child.

(1) childAddress=(MC∗addressParent)+1

The 6LoWPAN Ad hoc On-demand Distance vector routing (LOAD) is a simplified version of the well-known AODV developed for 6LoWPAN [45]. During the protocol functioning, it creates a mesh network topology without considering the IPv6 network layer. LOAD supports both the EUI-64 address and 16-bit short address. Seeking to reduce the size of control packets, LOAD does not use destination sequence numbers. Thus, to avoid loops, only the destination of an RREQ (Route Request) message can create an RREP (Route Reply) as a response. Although not mandatory, a LOAD node may use local repair when it detects a link break during data packet forwarding. When the local repair fails, a LOAD node may generate an RERR (Route Error) message to inform the originator of the data packet that it was not possible deliver the message to the destination. Different from AODV, an RERR message of LOAD indicates just one unreachable address. Due to limitations of network devices and seeking to reduce the computational resource usage, LOAD does not use a precursor list like in AODV. Furthermore, LOAD allows the use of the LQI (Link Quality Indicator) as a routing metric in addition to the hop distance. Although there are different ways of using LQI as a routing metric, LOAD adopts the LQI to avoid routes that have a link quality lower than an initially-defined threshold. Besides, LOAD does not use HELLO messages or passive acknowledgments.

In [46], the authors proposed a Dynamic MANET On-demand for 6LoWPAN (DYMO-low) routing protocol. DYMO-low is a routing protocol based on DYMO and, consequently, on the well-known AODV. DYMO-low uses three types of control messages: RREQ, RREP, and RRER (like in AODV). The protocol was fully projected for use over IEEE 802.15.4 devices. Thus, routing messages should not be fragmented. The choice of the best path to forward a message is done considering the LQI value and route cost. HELLO messages (used in AODV) are not used in DYMO-low. In contrast, the protocol uses IEEE 802.15.4 acknowledgment messages to determine if a neighboring node is reachable. Aiming to avoid loops, DYMO-low uses a simplified sequence number with 16-bits of length (DYMO sequence numbers are 32-bit). RREP must only be sent by the destination node of an RREQ. In DYMO-low, RERR messages indicate just one unreachable destination and are generated when: (i) an entry of the route table expires; (ii) a data packet cannot be successfully delivered to the next hop; or (iii) a broken link is detected.

A Hybrid Routing protocol for LLNs (HYDRO) was proposed in [47,48]. Hydrois a routing protocol for LLN, created to attend collection-based and point-to-point traffic. The hybrid approach combines local agility with centralized control. The network nodes create Directed Acyclic Graphs (DAGs) as default routes to the border router. On the other hand, the border router aggregates information about the nodes to create a global view of the network. This information is sent by the nodes through specific control messages or through opportunistic piggybacks in data packets. By creating a triangle between two nodes, the border router is responsible for forwarding the point-to-point traffic in the network. When a node wants to send a data message to another node in the network (point-to-point traffic), the packet is sent to the border router that should forward the packet to the desired destination. When an active point-to-point flow is detected, Hydro uses a special control message to optimize the message exchange between these two nodes. Thus, the data packets can be forwarded through the shortest paths without the use of a border router. Each node maintains statistical information about its neighbors. This information is used to measure link quality and assist in route selection.

These routing protocols were defined before the specification of the routing requirements of LLN (presented in Section 2). As they are only simplifications of already existent protocols, they were unable to meet the requirements identified by the RoLL working group. As already mentioned, after identifying that the existent routing protocols defined by IETF were unable to supply the demands of LLN, the RoLL working group started the development of RPL. A description of RPL is provided in the next subsection.

### 3.2. RPL

RPL is a tree-based routing protocol created by the RoLL working group and defined by IETF as the standard routing protocol for LLNs [24]. RPL organizes the network topology in a Destination-Oriented Graph (DAG), which is composed of one or more Destination-Oriented Directed Acyclic Graphs (DODAG). Each DODAG represents a routing tree created by a root node, also known as a sink node or LBR (LLN Border Router). To construct the DODAG, RPL uses the Objective Functions (OF) that adopt routing metrics to calculate the best path between nodes and the DODAG root [49]. Thus, the routing structure created by the RPL is a logical topology built on a physical topology according to the used OF.

In the RPL operation, the network nodes initially build the DODAG. Thus, in this first step, the root node starts the process of DODAG construction, sending a DIO (DODAG Information Object) message to its neighbors. This message carries various relevant information, such as node rank, mode of operation, OF, and metrics. Each node that receives a DIO message should process it and decide whether or not to join the DODAG according to the used OF [50]. If a node chooses to join the DODAG, it has an upward path to the root node. At this moment, the node computes its rank, refreshes its neighbor table, and chooses its preferred parent, which will be used to forward messages to the DODAG root. Usually, the preferred parent is the neighbor with the lowest rank computed according to the OF. Some nodes may be configured to provide routes to other nodes. If a node is set to be a router, it should update and resend the DIO messages to its neighbors. If the node does not resend DIO, it is defined as a leaf in the routing tree. Each node that receives the DIO message should process it and continue the operation until all network nodes can be reached [51].

RPL permits a new node to join the network at any time. In this case, the new node uses a DIS (DODAG Information Solicitation) message to request a DIO message of a node already incorporated in the DODAG. Through the reception of the DIO message, the new node selects its preferred parent according to the OF. Furthermore, a DIO message is used in DODAG maintenance. Based on the Trickle Timer [52], DIO messages are periodically sent by the nodes seeking to maintain network stability.

RPL allows both upward (from the node to its parent) and downward (from the node to its children) routes. Upward routes are naturally created during the initial process of DIO sending. However, the use of downward routes requires the handling of DAO (Destination Advertisement Object) messages. The DODAG nodes process the DAO messages according to the RPL Mode Of Operations (MOP), which are presented below. Independent of the MOP used, DAO messages may require reception confirmation, which should be done using DAO-ACK messages.

**Figure 1:**
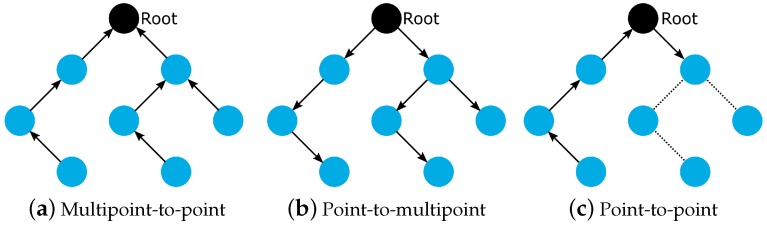
Traffic patterns supported by RPL. Lines with arrows indicate the traffic flow, while dotted lines without arrows indicate the links of the routing topology.

Although it is designed for the Multipoint-to-Point (MP2P) traffic pattern, RPL also admits the data forwarding using Point-to-Multipoint (P2MP) and Point-to-Point (P2P) [53]. In MP2P, the nodes send data messages to the root, creating an upward flow (Figure 1a). In P2MP, sometimes termed as multicast, the root sends data messages to the other nodes, producing a downward flow (Figure 1b). In P2P, a node sends messages to the other node (non-root) of DODAG; thus, both upward and downward forwarding may be required (Figure 1c).

**Figure 2:**
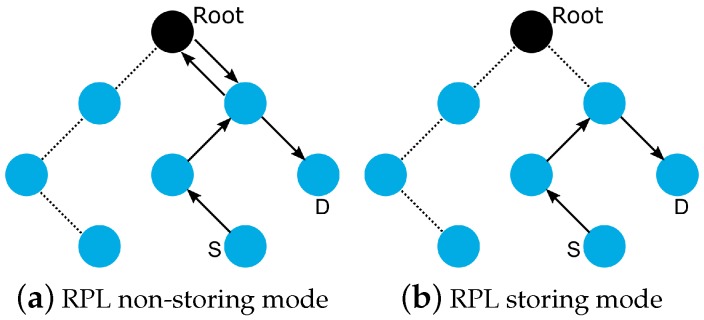
P2P message forwarding using Mode Of Operations 1 (MOP 1) and MOP 2. Lines with arrows indicates the traffic flow, while dotted lines without arrows indicate the links of routing topology

RPL defines four MOPs that should be used considering the traffic pattern required by the application and the computational capacity of the nodes. In the first, MOP 0, RPL does not maintain downward routes; thus, consequently, only MP2P traffic is enabled. In non-storing MOP (MOP 1), downward routes are supported, and the use of P2P and MP2P is allowed. However, all downward routes are maintained in the root node. Thus, the total downward traffic should be initially sent to the DODAG root and subsequently be forwarded to its destination as shown in Figure 2a. In storing without multicast MOP (MOP 2), downward routes are also supported, but are different from MOP 1; the nodes maintain, individually, a routing table constructed using DAO messages to provide downward traffic. Hence, downward forwarding occurs without the use of the root node, as illustrated in Figure 2b. Storing with multicast MOP (MOP 3) has a functioning similar to MOP 2 plus the possibility of multicast data sending. This type of transmission permits the non-root node to send messages to a group of nodes formed using multicast DAOs.

In RPL, the whole process of DODAG construction is highly dependent on the OF considered by the network. Furthermore, the network performance is directly linked to the process of route selection. According to the metrics and constraints applied to select the best path between a node and a root, it is possible to increase or decrease the network performance. Furthermore, as already explained, some applications require different performance of the routing protocol. For example, e-health applications need low latency and high reliability [54]. On the other hand, some applications may require low energy consumption and mobility, such as animal monitoring applications. Thus, different types of applications can require different OFs. To fulfill the application requirements, each OF should contemplate metrics or constraints that better attend to these conditions. Based on the information of OFs, the node computes the weight of each path to the root. Hence, the selection of preferred parent and the rank calculation are performed according to OF. IETF defines, as the standard, two different objective functions: Objective Function zero (OF0) and Minimum Rank with Hysteresis Objective Function (MRHOF).

Objective Function zero (OF0) [55] is defined in the RFC 6552 and is designed to find the shortest path to the root. Using the DOI message information, OF0 knows the rank of each candidate neighbor. OF0 selects, as the preferred parent, the candidate neighbor with the best rank (the shortest). Additionally, OF0 stores a backup feasible successor to use as an alternative to the preferred parent. All the upward traffic received by the node is routed to the root using the preferred parent or the backup successor, according to the link conditions. OF0 does not attempt to perform any load balancing. The backup successor is used to forward a packet to the root node when it is not possible to use the preferred parent. The rank of a node is obtained through the sum of the rank of its preferred parent and the value of rank_Increase, which is computed using a specific equation and predefined variables [55].

The Minimum Rank with Hysteresis Objective Function (MRHOF) [56] was developed to select the path with the lowest path cost without promoting excessive changes of the preferred parent. Thus, MRHOF uses two mechanisms to fulfill this purpose. The first seeks to find the path with minimum cost, while the second performs the hysteresis. Using the hysteresis mechanism, a candidate parent is selected as the preferred parent only when it has a path cost smaller than the current preferred parent, minus a given threshold [57]. Usually, MRHOF uses the ETXrouting metric to compute the cost of each path. However, it can utilize any additive routing metric described in [58]. During the network functioning, the path cost is re-computed periodically. Nevertheless, the preferred parent selection process is executed just when the path cost for a neighbor is refreshed or a new neighbor is inserted in the neighbors’ table. As mentioned previously, the replacement of the preferred parent needs to attend to the hysteresis condition to avoid constant parental changes.

### 3.3. LOADng

The LOADng (Lightweight On-demand Ad hoc Distance-vector routing protocol-next generation) is a reactive routing protocol based on AODV and adapted for LLNs. LOADng was presented in an Internet-Draft to IETF in October 2011 [59]. Since then, LOADng has received various updates, and its last version was published in July 2016 [60]. Similar to AODV, LOADng only creates a route when a node needs to send a data message to another node. The process of route creation (also known as route discovery) is performed through the use of control messages adapted from AODV. The RREQ (Route Request) message is used during the route discovery process to find a path to the desired destination. The RREP (Route Reply) message is used by the destination of an RREQ to attend to the received route creation request. To allow the creation of bi-directional paths, an RREP can require a reception confirmation. In this case, an RREP_ACK (Route Reply Acknowledgment) message is used to answer the sender of the received RREP. Further, the LOADng also can use RERR (Route Error) messages to inform problems detected during the data message forwarding. In general, RERR is used when an intermediate node does not know the destination of a data message.

During the route discovery process of LOADng, the nodes should store a set of information about the other nodes of the network. Thus, each node has a routing set, a pending acknowledgment set, and a blacklisted neighbor set. The routing set, which works as a routing table, is formed by a set of entries created during the route discovery process. Each entry of the routing set contains information such as the destination address of the route, the address of the next hop to the destination, and the number of hops to reach the destination. The pending acknowledgment set is used to store data about the RREP messages sent with the requirement of an acknowledgment. As a complement to this last, the blacklisted neighbor set is employed to maintain the address of the nodes that have not replied with an RREP that required a reply (an RREP_ACK).

**Figure 3:**
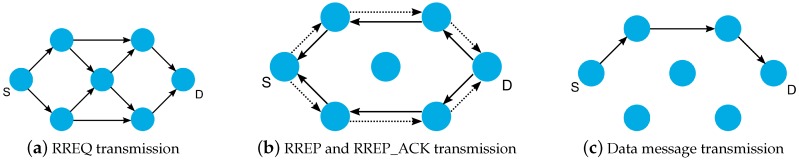
LOADng control message transmissions. Arrows indicate the message flow. (**a**) Transmission of Route Request (RREQ) messages flooding all network nodes. (**b**) Transmission of Route Reply (RREP) and RREP_ACK messages, where bold lines indicate RREP messages that are forwarded through the path constructed by RREQ and dotted lines indicate RREP_ACK messages that are optionally sent after reception of each RREP, but not routed. (**c**) Transmission of data messages through the path selected by Snode after receiving RREP.

In the protocol functioning, when a node *S* needs to send a data message to a destination *D*, it first verifies its routing set, seeking a path to the desired destination. If the route is found, the node should use it to send the message. Otherwise, *S* must start the route discovery process. Thus, *S* creates an RREQ message with the same destination as the data message (node *D*). In sequence, *S* broadcasts the RREQ message to all its neighbors. Each node that receives the RREQ should verify several fields to identify whether it is valid for processing. This verification also avoids the creation of loops. The information carried in the message is used to update the routing set of nodes. In the sequence, the node refreshes the message fields and, if necessary, forwards it to the other nodes using broadcast transmission. The RREQ message is forwarded by the intermediate nodes until reaching the destination *D* (Figure 3a). When *D* receives the RREQ, an RREP message is generated and sent to reply to the request originated by *S*. The RREP is forwarded in unicast using the path stored in the routing set during the RREQ broadcasts. If required by *D*, each intermediate node that receives the RREP should send an RREP_ACK to the previous hop to confirm the RREP reception (Figure 3b). When *S* receives the RREP, the route discovery process is finished and the data message can be sent through the created path (Figure 3c).

The RREQ and RREP messages carry fields to inform about the hop count and hop limit. Thus, so that these messages are always received, the node should increment the hop count and decrement the hop limit. The hop count field is used to inform about the number of hops traveled by the message since its originator. The hop limit field is implemented to indicate the number of remaining transmissions of the message. Thus, when a message is received with the hop limit equal to zero, it should not be forwarded to other nodes. Both messages also can transport another routing metric that should be computed and updated according to its specifications.

As mentioned above, LOADng is based on the well-known AODV. However, to permit the protocol to be executed on devices with severe hardware restrictions, some simplifications were made. In the LOADng, the routing table does not store the whole path to reach a destination. Besides, the control messages do not carry the address of each node traveled. Furthermore, only the destination of an RREQ message can reply to it using an RREP. Thus, the use of “intermediate RREP” mechanisms is not allowed. Finally, LOADng permits the use of a wide variety of addressing schemes (e.g., IPv6, IPv4, and Rime), and allows the use of different routing metrics as an alternative to the hop count.

## 4. Enhanced Routing Solutions for Internet of Things Networks

**Figure 4:**
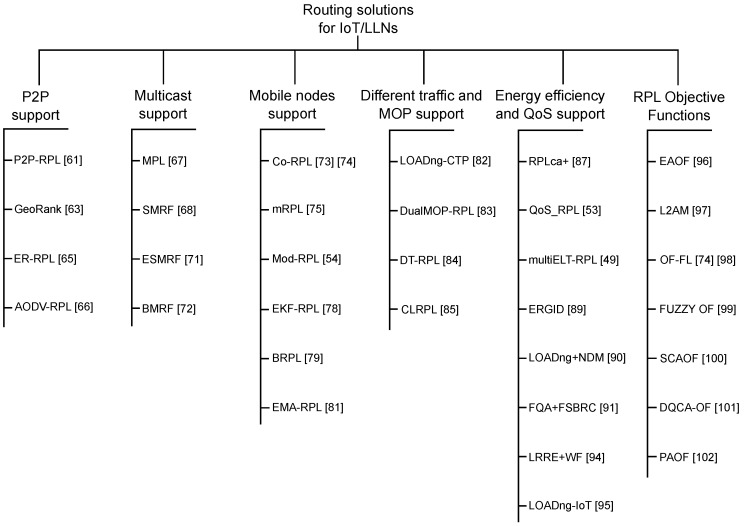
Taxonomy of routing solutions for IoT/Low-power and Lossy Networks (LLNs). ER, Energy-efficient Region-based; AODV, Ad-hoc On-demand Distance Vector; MPL, Multicast Protocol for Low-power and lossy networks; ESMRF, Enhanced Stateless Multicast RPL Forwarding for IPv6-based low-lower and lossy networks; BMRF, Bidirectional Multicast RPL Forwarding; BRPL, Backpressure RPL; DT, Diverse Traffic; CLRPL, Context-aware and Load balancing RPL; ELT, Expected Lifetime; ERGID, Emergency Response IoT based on Global Information Decision; NDM, Neighbor Disjoint Multipath; FQA, Fuzzy Q-Algorithm; FSBRC, Fuzzy System-Based Route Classifier; WF, Weighed Forwarding; EAOF, Energy-Aware Objective Function; OF-FL, Objective Function Fuzzy Logic; SCAOF, Scalable Context-Aware Objective Function; DQCA, Delivery Quality- and Context-Aware; PAOF, Parent-Aware Objective Function; Co, Corona.

Currently, the routing solutions proposed for IoT/LLN are performed with the improvement of existent and well-known base protocols. Although several protocols have been used as inspiration, the principal and most recent approaches are based on the RPL and LOADng. Figure 4 presents a taxonomy of studied routing solutions, divided according to their proposals and motivation. Each considered solution is presented and described in the following subsections. Furthermore, at the end of each subsection, a summary table is shown to present the highlights of each approach, such as base protocol, objectives, descriptions, strengths, weaknesses, and application scenarios. The objective column presents the aims of the routing solution. The description column shows the method used by the proposal to reach its objectives. The strengths and weaknesses columns present, respectively, the main contributions and limitations of the solution. Finally, the application column shows the type of application to which the solution should, or intends to, be applied.

### 4.1. P2P Communication Support

**Figure 5:**
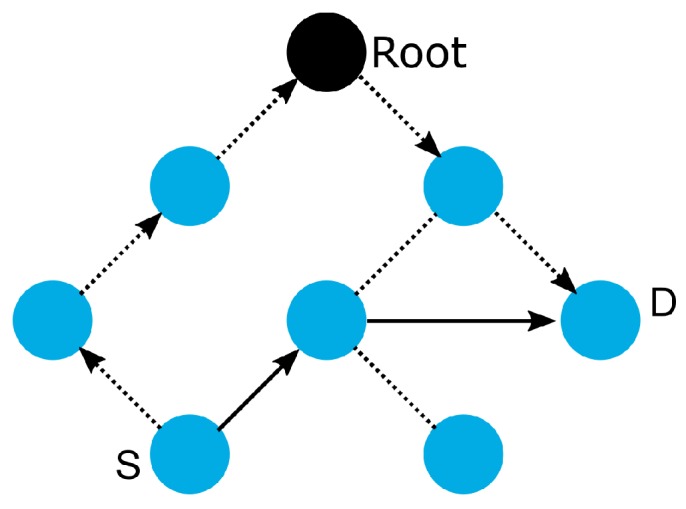
Transmission of a P2P message from node S to node D. The dotted lines with an arrow represent the path created by RPL, while the solid lines with an arrow represent the path established by P2P-RPL.

Peer-to-peer communication represents an import traffic pattern in LLNs, mainly in IoT scenarios. Although the default RPL implementation supports P2P traffic, the approach introduced by the IETF standard can be considered very costly in some situations. Thus, in April 2010, RoLL WG started the proposal of a new solution to improve the P2P supported by RPL. Hence, in August 2013, the Reactive Discovery of Point-to-Point Routes in Low-Power and Lossy Networks (P2P-RPL) was presented as an IETF standard in the RFC 6997 [61]. P2P-RPL is an extension of RPL that allows the creation of P2P routes on demand, working as a reactive routing protocol. Thus, a source node that wants to send a P2P data message should start a route discovery process, creating a temporary routing tree. The source, which acts as the root of the temporary DODAG, sends a P2P Route Discovery Option (P2P-RDO) across the entire network requesting a route to the destination node. Each node that receives the P2P-RDO must first check the message destination, determine if it joins in the DODAG, and select its temporary preferred parent. Furthermore, if the node is not the destination, it should forward the P2P-RDO. The P2P-RDO is forwarded across the network until it reaches its destination node or accomplishes its maximum number of hops. When the destination receives the P2P-RDO, it must generate a P2P Discovery Reply Object (P2P-DRO) to answer the route request. The P2P-DRO is forwarded back through the preferred parent chosen during the P2P-RDO forwarding. The intermediate nodes should store the reverse route used to deliver the P2P-DRO for a future data message forwarding. The source of P2P-RDO, after receiving the P2P-DRO, should start the sending of the P2P data message through the route created. Complementarily, P2P-RPL permits the use of a mechanism to verify the necessity of starting a route discovery for P2P data sending. This mechanism, introduced in the RFC 6998 [62], uses Measurement Objects (MO) that assess the quality of the P2P route created by the default RPL. Whether or not it can fulfill the application routing requirements, the node should use it. Otherwise, the node should initiate the process of P2P route discovery (as previously described) seeking to attend to the application constraints. Figure 5 exemplifies the routes that can be created using both RPL and P2P-RPL for the transmission of a P2P data message.

A geographic routing approach for 6LoWPAN was presented in [63]. The proposed GeoRank is based on RPL and GOAFR (Greedy Other Adaptive Face Routing) [64]. The GOAFR algorithm is able to seek optimal or sub-optimal routes in networks with high link density. However, considering networks with low link density, RPL can demonstrate a better performance than GOAFR. Thus, seeking to mix the better of two approaches, the authors proposed GeoRank. The approach aims to improve the P2P support of 6LoWPAN and reduce the amount of control messages required in this type of traffic. In its operation, the GeoRank algorithm initially calculates the distance between the source node and the destination based on the list of DODAG roots. After, the root with the lowest absolute angle difference between the source and the destination is selected as the anchor node. Subsequently, the algorithm tries to perform the routing of packet using greedy forwarding. In this mode, the node holding the message tries to send it to its neighbor with one hop to reach the message destination. If this is not possible, the algorithm assumes the GeoRank mode and then forwards the packet to the preferred parent in the path to arrive at the selected anchor. This forwarding process should occur until the message reaches a node closer to the destination than the anchor node. If this condition is attained, the greedy forwarding mode is assumed again. Otherwise, the message reaches the anchor and is forwarded in the face routing mode of GOAFR until it comes to its destination. In GeoRank, due to the use of geometric calculation, all nodes of the network must be static or be equipped with GPS. The use of mobile nodes is possible when they can communicate with a static node using just one hop. Furthermore, the message to be routed must store information about the position of its anchor. Although the authors used smart lighting system as an example of application for using GeoRank, they highlighted that the proposed solution might not meet the requirements of this kind of application.

Considering that the majority of P2P routing protocols, including P2P-RPL, flood the network with control packets to create routes, provoking great overhead and energy consumption, Zhao et al. [65] proposed the Energy-efficient Region-based Routing Protocol (ER-RPL). The approach aims to allow an energy-efficient P2P communication without harming the network’s reliability. ER-RPL presents a hybrid approach, mixing proactive and reactive features based on RPL. The main idea of the protocol is to segment the network into different regions and realize the P2P route discovery, just considering the areas in which the nodes are located. To this end, ER-RPL requires the existence of location-aware nodes (e.g., equipped with GPS) in the network, named Reference Nodes (RN). The protocol uses the coordinates of RNs to partition the network into different regions and to calculate the distance between nodes and RNs. Afterwards, the nodes, based on the distance to RNs, should select their regions. A binary number, named the Region Code (RC), identifies each region. This process occurs in an initial stage of the protocol when the RNs floods the network with Region Formation Object (RFO) control messages. For a P2P communication, the source node first should send a P2P route request to the destination node using the root node, i.e., the default route of RPL. This request is made using a control message named Message Request Object (MRO). After receiving the MRO, the destination node should verify whether the existent path is satisfactory based on the route cost. If valid, the destination node, through the reverse path and using an MRO, should inform the source node to start the sending of data. Otherwise, the destination should initiate the process of region-based route discovery. In this process, the destination node uses MRO to create a temporary DODAG just considering the regions between it and the source. Subsequently, this DODAG is used to send the data packets. Note that, at this stage, RN does not realize any task. Thus, ER-RPL functioning, although complex, can reduce the network energy consumption by avoiding the flooding of the whole network with the P2P route discovery packet. Further, the approach allows the sending of P2P messages without the use of a root node, creating near-optimal paths that can fulfill the reliability requirements of applications. For the other traffic patterns, such as MP2P, ER-RPL uses the structure built by default RPL functioning.

Although P2P-RPL can allow improved P2P data traffic for RPL networks, it still lacks in some areas. The protocol admits that the links among the nodes are symmetric, i.e., the quality of the linkage from Node A to Node B is the same (or almost the same) from B to A. Hence, the P2P messages exchanged between these nodes use a single path. However, in a practical environment, this symmetry may not be reached and, consequently, may not satisfy the routing requirements of a large variety of IoT applications. Thus, the Ad hoc On-demand Distance Vector routing-based RPL (AODV-RPL) [66] emerges as an improvement of RPL to support the P2P traffic pattern considering both the symmetric and asymmetric link during the path discovery process. As P2P-RPL, the peer-to-peer routes in AODV-RPL are constructed on-demand. A source node begins the route creation process to a destination when the current path does not fulfill the application requirements or the desired path does not exist. Thus, the source node must send DIO-RREQ (DIO with Route Request content) messages to create an RREQ-instance (DODAG created by the originator of an RREQ message) and find a path to the desired destination node. DIO-RREQ has a field to carry information about whether the route is symmetric or asymmetric (*S* field). Each node that receives the DIO-RREQ must verify the condition of the bidirectional link, update the *S* field, if necessary, and choose whether to join in the RREQ-instance. In this case, if a link is set as asymmetric, all routes that use this link are considered asymmetric. When DIO-RREQ reaches its destination, the node, based on the *S* field, must choose how to answer the route creation request. The destination node can also wait for a predefined period to receive DIO-RREQs through other routes. If the *S* field of the message indicates that the route created is symmetric, the destination node should unicast a DIO-RREP (DIO with Route Reply content) to the DIO-RREQ originator through the created path. Therefore, due to being symmetric, this single path can be used for data transportation in both directions. Otherwise, in case the route created is asymmetric, the destination node should start the creation of an RREP-instance (DODAG created by the destination of an RREQ message) multicasting DIO-RREP to its neighbors. DIO-RREPs are forwarded realizing the link symmetry verification, similarly to DIO-RREQs, until reaching the DIO-RREQ originator that, after receiving it, should initiate the sending of data messages through the path created by the DIO-RREP. Notice that when AODV-RPL does not find a symmetric path between two nodes, it can create a pair of DODAGs (RREQ-instance and RREP-instance) with a single-route discovery process. Thus, the P2P data message exchange can be performed using two different paths: a route from A to B and another from B to A. However, AODV-RPL is still an IETF Internet-Draft and can suffer modifications until its complete definition. Furthermore, to the best of the authors’ knowledge, no other paper in the literature realizes a performance evaluation study about the efficiency of AODV-RPL.

Table 1 presents a summary of the above-described routing solutions.

### 4.2. Multicast Communication Support

Multicast communication represents a fundamental transmission type for several IoT applications. As pointed out in Section 2, an industrial environment application running over an LLN can require the sending of alert messages for a predefined group of nodes or base stations. To achieve this, the routing protocol should realize reliable multicast transmissions.

Proposed in 2010, the Multicast Protocol for Low-power and lossy networks (MPL) [67], which was introduced in parallel to RPL and initially called trickle multicast, was the first effort of IETF to provide IPv6 multicast support for LLN. Defined as an IETF standard in 2016 through RFC 7731, the MPL uses a flooding mechanism controlled by trickle timers [52] to perform multicast message sending without the necessity to maintain routing tables. Although the MPL is an independent protocol, its solution is sometimes considered an extension to provide multicast support for RPL. In MPL, all network nodes can receive a multicast message depending on whether they are inserted in a multicast group. As all nodes can broadcast the received multicast packets, the implementation of a mechanism for avoiding duplicated messages is necessary. Thus, MPL includes a sequence number in the packet header for distinguishing each multicast message, besides storing the most recently-received messages in a buffer. After receiving a multicast message, the node should verify whether it was already received by checking its sequence number or presence in the buffer. If the node identifying the message is already received, it should discard it. Otherwise, the node must record the message sequence number, store it in the buffer, process the payload, and multicast it to the other nodes. MPL uses trickle to organize both control and data messages exchanged among the nodes. The nodes can use control messages for informing their neighbors about their set of sequence numbers or buffer. When a node identifies that a neighbor has not yet received a message, it redefines its trickle interval to the minimum value to quickly disseminate the packet missed by the neighbor. Thus, due to storing the most recent packets in a buffer and to allow local retransmission of lost packets, MPL can offer high reliability for the packet delivery ratio.

**Figure 6:**
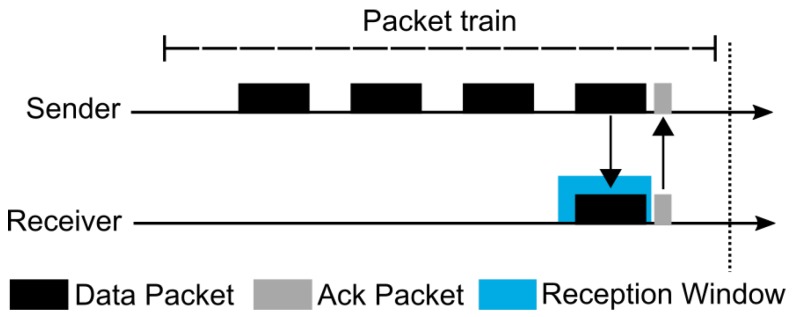
ContikiMAC unicast packet transmission.

**Figure 7:**
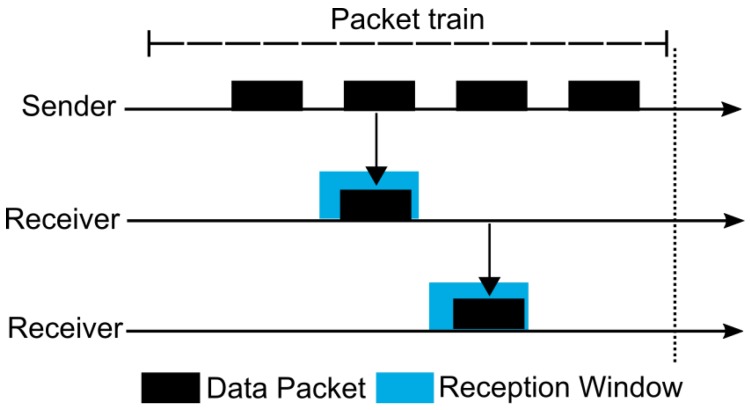
ContikiMAC broadcast packet transmission.

**Figure 8:**
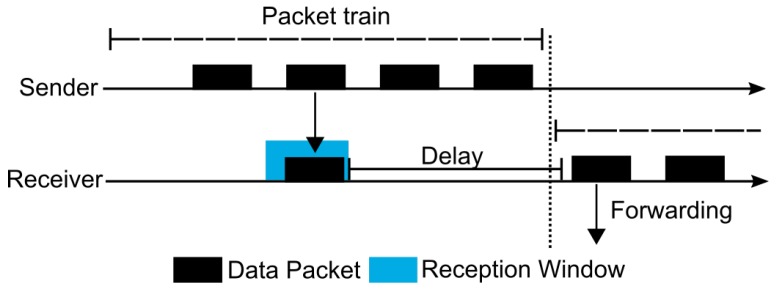
SMRF mechanism for multicast packet transmission.

Although MPL can provide high reliability, it can also present a high end-to-end latency and communication overhead. Thus, trying to mitigate these drawbacks, Oikonomou et al. proposed the Stateless Multicast RPL Forwarding (SMRF) [68]. The proposal was constructed over RPL and uses the “storing mode of operation with multicast support” (multicast RPL or MOP 3) of the IETF standard solution to offer enhanced results when compared with the default approach. SMRF proposes a cross-layer mechanism that improves the functioning of Radio Duty Cycling (RDC) protocols for multicast forwarding operations. As an example, the authors cited ContikiMAC [69], a duty cycling mechanism present in the Contiki OS [70] and widely used in IoT/6LoWPAN networks. ContikiMAC presents low radio usage for reducing the power consumption of the nodes. Thus, the radio spends the majority of its time sleeping (turned off), waking-up (turning on), and periodically trying to identify a possible packet transmission. The duration of the period during which the radio is turned on is called CCI (Channel Check Interval). A node, to send a message, realizes several transmissions of the same packet during a time interval intending to reach an awake receiver. This repetitive transmission is referred to as a packet train. In a unicast transmission (Figure 6), a receiver node detects a packet sent during its CCI and starts the reception window until it receives the whole message. Afterwards, the receiver must send an acknowledgment to the sender, which finishes the packet train transmission. Differently, during a broadcast transmission (Figure 7), the sender must send a message during the entire packet train interval. Note that, due to a possible disturbance in the communication of other nodes, the transmission of acknowledgment packets is not used in ContikiMAC broadcast transmission. A receiver node cannot immediately forward the received packet due to possible collisions with the sender that may still be transmitting. For that reason, SMRF proposes the use of a short delay between the message reception and its forwarding for allowing the use of ContikiMAC broadcast and avoiding possible collisions (Figure 8). This behavior prevents the use of multiple ContikiMAC unicasts to the task of multicast transmission, commonly used by multicast RPL, and contributing to the reduction of energy consumption. Furthermore, multicast RPL does not specify any control mechanism to avoid the reception and transmission of duplicated multicast packets. Thus, SMRF constrains that a node must only process multicast packets received from its preferred parents.

SMRF, although showing several benefits when compared with standard RPL multicast, still presents some limitations. One of the main drawbacks of SMRF is the lack of support for upward multicast. Due to its mechanism for avoiding the processing of duplicated packets, as mentioned above, SMRF only allows nodes to process packets sent from its preferred parent. Thus, messages received from children nodes are neither processed nor routed. Therefore, to allow the nodes to transmit multicast messages both upward and downward, the Enhanced Stateless Multicast RPL Forwarding for IPv6-based low-lower and lossy networks (ESMRF) was proposed [71]. ESMRF proposes that multicast messages are first sent to the root node that, afterwards, should realize the message forwarding to its final destination. Thus, when a node wants to send a multicast message, it should encapsulate its content and destinations in an ICMPv6 (Internet Control Message Protocol Version 6) packet. This packet, also named a delegation packet, is sent in unicast to the root node (using the default RPL upward mechanism), which is in the top of the tree structure and can communicate with all network nodes. After receiving the delegation packet, the root node must extract the multicast content and send it to the destinations indicated. The process of forwarding multicast messages performed by the root node is similar to that proposed by SMRF. Thus, by using the same principle of SMRF, ESMRF also avoids the processing of duplicated packets.

ESMRF allows, using a simple approach, both upward and downward multicast communication, solving the gap of SMRF. However, the proposed solution suggests the use of the root node to forward all multicast messages. This approach becomes too expensive in a large routing tree, provoking communication overhead and high end-to-end latency. Thus, to mitigate the problem found in SMRF with a more robust method than proposed by ESMRF, Lorente et al. [72] introduced a new multicast routing protocol named Bidirectional Multicast RPL Forwarding (BMRF). BMRF seeks to merge the best features of RPL and SMRF. Thus, the approach offers three different modes for packet sending. In unicast mode, the sender node verifies the nodes interested in the multicast message according to the routing table, and then, the message is sent in unicast to each one, as proposed by RPL multicast. In broadcast mode, the sender verifies the group of nodes that want to receive the multicast message, and then, the communication is performed as in the broadcast proposed by SMRF. In the last mode, mixed-Tmode, the sender performs the transmission according to a *T* threshold that decides between multiple unicasts or broadcasts. *T* is defined according to the number of children interested in the message and the RDC rate.

During BMRF functioning, when a node wants to send a multicast message, it sends the packet both upwards and downwards, except for the root node, which should not be sent upwards, and leaf nodes, which should not be sent downwards. The upward sending is performed using unicast transmission. Otherwise, the downward sending is performed according to the BMRF mode. This behavior is necessary for avoiding duplicated packets. After receiving a multicast message, a node can process it depending on whether the packet was received from above or below. If the message was received from above, the node just should process it if the sender is its preferred parent (checked via MAC address). The node also consults its interest in the message to send it to the upper layer and verifies its routing table to forward the message to its children nodes. If the message was received in broadcast, the node should insert a delay in forwarding it (as in SMRF). This forwarding should be done according to the BMRF mode selected. If the multicast message was received from below and indicated with a unicast MAC address, the node should process the message once it was sent in unicast by a child node. Thus, the receiver node checks its interest in the message and then forwards it in unicast to the children inserted into the multicast group. The node also sends the message upwards, in unicast, if it is not the root. Hence, BMRF allows all network nodes to send and receive multicast messages, regardless of its position in the routing tree. Additionally, BMRF supports dynamic multicast group registration and avoids delivery disorder.

A summary of routing solutions for multicast support described in this subsection is presented in Table 2.

### 4.3. Mobile Nodes’ Support

In addition multicast transmission, the support for mobile nodes also represents an important task for the majority of applications in IoT environments, mainly in urban and industrial scenarios. Thus, an efficient mobility support protocol should provide fast, continuous, and reliable communication among mobile and static nodes. Hence, in [73,74], the authors presented a variation of RPL based on the Corona mechanism (Co-RPL), which aims to ensure the quality of service in LLNs with mobile nodes. Co-RPL uses the default RPL control messages with additional fields to fulfill its purpose. All network nodes maintain a table with information about their neighbors (ID, DAG, corona, and link quality). When a node receives a DIO message, it selects its best parent, defines its corona ID, and computes its rank based on the objective function. The corona ID is determined through the result of the sum of the lowest corona ID among its neighbors plus one. After updating the DIO, the node resends the message in a broadcast. If a router node receives other DIO messages, it must select the preferred parent among the neighbors with the lowest corona ID. Whenever the corona ID of a neighbor changes or a new neighbor is detected, the node should execute the neighbor discovery process to update the corona IDs. Co-RPL proposes a new path recovery mechanism. When a node cannot reach the message destination, it sends the data packet to any node of the upper corona ID and sends a DIS message to its children to pause the sending of the data packet. This process occurs until the router node finds a new preferred parent. If a node cannot send the data packet to any neighbor of the high corona ID, it must inform the previous hop to pause the sending of data packets.

Fotouhi et at. [75] proposed the mRPL, a solution to enhance the mobility support in RPL. The proposed solution integrates the default RPL with smart-HOP [76,77], a hands-off mechanism initially developed for WSN. In mRPL, smart-HOP uses RPL-control messages like beacons. Considering the existence of two different types of nodes, which are Mobile Nodes (MN) and serving Access Points (AP), the smart-HOP mechanism is divided into two phases. In the data transmission phase, an MN sends a sequence of *n* DIS beacons to a serving AP. After receiving the *n* DIS, the serving AP calculates the Average Received Signal Strength Indication (ARSSI) value based on the *n* received messages and sends the obtained value to MN inside a DIO message. If the MN detects that the received ARSSI is lower than a defined threshold, the discovery phase begins. In this phase, an MN sends DIS control messages periodically until it receives a DIO message from an AP with an ARSSI greater than the defined threshold. When this condition is fulfilled, the MN returns to the data transmission phase with a new preferred parent. An MN can also start the discovery phase after detecting that its serving AP parent is not sending DIO messages. It is important to note that the messages exchanged in these two phases are disassociated from the data messages and control packets of RPL. Thus, mRPL nodes can coexist and interoperate in the same network when nodes execute default RPL.

Considering the necessity of mobility support for healthcare and medical applications, Gara et al. [54] proposed an adaption of RPL named mod-RPL. The approach contemplates applications executed over networks containing both mobile and static nodes. Thus, to attend to the routing requirements, the mod-RPL configures all mobile nodes as leaves of the DAG created by RPL. Therefore, mobile nodes are unable to send DIO messages and, consequently, be a parent of other nodes or perform routing tasks. The operation of the static nodes is equal to the default RPL. However, mobile nodes must not create or forward DIO messages, avoiding the creation of sub-DODAG and connection loss with possible child nodes. All the others’ operations performed by mobile nodes (DIS and DAO sending, parent selection, and rank computation) are done like in the default RPL. In the end, mod-RPL regulates the transmission of control messages in the same way as the default RPL considering that the mobility in healthcare applications is not high.

Another routing solution for supporting mobile nodes in LLNs was proposed by Bouaziz et al. in [78]. Based on RPL, the Extended Kalman Filter for Mobile RPL (EKR-MRPL) aims to reduce the signaling overhead and energy consumption caused by constant changes of attachment points of mobile nodes. EKF-MRPL assumes that MNs do not execute any routing task. Thus, MNs just send and receive data from their preferred parent. EKF-MRPL operates following the basic concepts of RPL to construct the routing tree and execute the message forwarding. However, the new approach gives particular attention to the movement of MNs. This proposal is divided into three phases: movement detection, reaction, and notification. During the first phase, when MN is sending data messages to its Preferred Parent (PP), the PP should compute the RSSI for each packet received. Whether or not the RSSI value degrades below a predefined threshold, PP identifies that MN is moving away and should indicate it to find a new association node, i.e., a new PP. The current PP realizes this indication using a DIS message (flag = 1). After receiving the indication, MN starts the second phase. During this phase, MN should consider its direction and neighbor list to choose the new PP that can offer greater linkage time. For this purpose, MN broadcasts DIS (flag = 2) messages requesting the reception of DIO messages (flag = 1) from its static neighbors. After receiving DIO messages, MN should compute the RSSI of each neighbor. The computed RSSIs are used by the Extended Kalman Filter (EKF) to predict the MN movement trajectory. Posteriorly, MN should select its new PP considering its future movement and start the third phase. In this last phase, MN should send a DAO message to notify its neighbor that it was selected as the new PP. Furthermore, if possible, the MN should transmit a DAO “no-path” message to its previous PP to inform the disconnection. Notice that, between Phases 2 and 3, the MN can stop the sending of data messages to avoid packet loss and energy consumption. Hence, EKF-MRPL, through its movement prediction mechanism that allows MNs to select PPs with greater linkage time, reduces the number of preferred parent changes, contributing to the reduction of control message usage. Consequently, the protocol helps to reduce energy consumption and increase packet delivery.

Aiming to provide mobile support to LLN with highly dynamic network traffic, Tahir et al. proposed the Backpressure RPL (BRPL) [79]. BRPL, according to the authors, is the first routing protocol for LLN that merges RPL and the concepts of backpressure routing [80] to provide support to mobility and adaptability to various throughputs. As RPL, BRPL allows the use of multiple logical topologies (multiple DAGs) that can be created according to different OFs. However, contrary to RPL, each BRPL node maintains a queue of buffered packets for each DAG. Information such as RPL rank, queue length, and maximum queue length are exchanged among the nodes using DIO messages. Thus, each node that received a DIO message should update the information about the packet sender in the neighbor table. In the packet forwarding, the link weight of each neighbor is computed considering both its rank value and queue length. BRPL also uses a θ (theta) parameter to represent the tradeoff among the variables employed in the link cost computation (length of queues and rank of nodes). The θ value, which varies between zero and one, is dynamically adjusted by the QuickThetaalgorithm. QuickTheta defines the θ value according to the congestion level and the mobility of nodes. The congestion level is obtained by the ratio between the queue length of the node and its maximum queue. The mobility of the node, defined as β and computed by QuickBeta, is calculated in accordance with the changes in the neighbor table. Thus, the θ value is automatically adjusted for different traffic loads and topology changes provoked by mobility. θ also defines the switching of the routing strategy of BRPL. When its value is equal to one, BRPL realizes the packet forwarding using only the objective function of RPL. Otherwise, when θ is nearest to zero, BRPL tends to perform the forwarding task inspired by backpressure routing concepts. This dynamic strategy allows BRPL to achieve a significant packet loss reduction as detriment of an increased end-to-end delay.

An energy-efficient and mobility-aware routing protocol based on RPL was presented in [81]. The proposal, named EMA-RPL, considers low power networks composed by static nodes and MNs. To avoid the broken routes caused by the nodes’ movement, the MNs are always defined as a leaf in the RPL tree. This feature prohibits MNs from performing the data message routing received from other nodes. MNs send and receive data messages through a static node, named the Associated Node (AN). Moreover, an AN is responsible for monitoring, predicting the movement, and defining a new AN to the MNs. The EMA-RPL functioning is divided into three different phases: mobility detection, reaction and prediction, and notification. The first phase is started when an MN moves away from its AN. The AN performs monitoring and detects the movement of MN nodes based on the RSSI value measured when data messages are received. If the measured RSSI is lower than a predefined threshold, the reaction and prediction phase is triggered. In this phase, the AN sends control messages searching for a new static node able to be used as the AN of the MN. When the new AN is found, the notification phase is started to inform the MN. Thus, the previous AN sends a control message to the MN informing about the change. After receiving this message, the MN should confirm the message reception and perform the AN change on its routing table. By avoiding MN sending several control messages monitoring its movements, EMA-RPL can reduce the energy and computational resource usage of mobile devices. However, in a dense and high mobility scenario, this approach can overload the static nodes and reduce the network performance. Furthermore, the proposal requires several changes in the structure of RPL control messages, which can make the interoperability of the EMA-RPL difficult with other approaches and the standard RPL implementation.

Table 3 presents a summary of the above-mentioned routing solutions for mobile nodes’ support.

### 4.4. Different Traffic and Mode of Operations’ Support

IoT/LLN applications can require the use of different data traffic patterns during its execution. As an example, two nodes can exchange messages among them creating P2P traffic, and a few moments later, both need to send data to a central node generating MP2P traffic. Furthermore, the central node can send data messages to a specific group of nodes using multicast communication (or P2MP). Thus, based on this requirement, the routing protocols should provide support for different traffic patterns, preferentially permitting that all these can be used simultaneously.

The main feature of LOADng is P2P traffic support. However, the most significant limitation of the protocol is the lack of efficient support for MP2P and P2MP. Seeking to reduce this drawback, Yi and Clausen [82] proposed the LOADng Collection Tree Extension (LOADng-CTP). The introduced improvement allows the LOADng to function as a proactive routing protocol and to perform the creation of a routing tree for the collection of data messages from the leaf nodes to the root (similar to what happens in the RPL). The LOADng-CTP introduces two new flags on the RREQ messages (RREQ_TRIGGER and RREQ_BUILD) and a new HELLO message. At the begin of protocol functioning, the root node floods the network using an RREQ_TRIGGER message (RREQ with the RREQ_TRIGGER flag set to true) to indicate the interest in constructing the routing tree. The nodes that receive the RREQ_TRIGGER answer it using the HELLO messages. Each receiver of a HELLO should set the message sender as bidirectional on its routing set. After a predefined time, the root node floods the network again using an RREQ_BUILD message (RREQ with the RREQ_BUILD flag set to true). The receivers of an RREQ_BUILD verify whether the message was received from a neighbor set as bidirectional, and if true, a new route entry to the root is inserted in the routing set. After this procedure, the collection tree is built, and the upward traffic (MP2P) can be performed more efficiently. Optionally, LOADng-CTP also permits the construction of a reverse path to facilitate the downward traffic. For this, each node that receives an RREQ_BUILD should answer it using an RREP message. Thus, LOADng-CTP overcomes one of the main limitations of LOADng, allowing it to support different traffic patterns appropriately.

On the RPL, the supported traffic pattern is defined according to the utilized MOP, as described in Section 3.2. However, the choice of ideal MOP to be adopted also involves the consideration of the computational resources of the devices. Choosing the MOP with support to different traffic patterns (i.e., storing MOP) can require more hardware capacity and prevent “weaker” devices from running the protocol. In this case, a more reasonable solution could be to create a network with a different MOP being executed by the nodes with different capacities and making the “weaker” nodes work only as leaves. However, the presented scenario could face several interoperability problems once control messages are used and processed differently according to the MOP. Furthermore, as indicated on the RPL official document, each MOP should be adopted individually by the whole network. Thus, considering the limitations that may emerge for the using of different MOP simultaneously, Ko et al. have presented the DualMOP-RPL [83], an improved version of RPL that supports both storing and non-storing MOP at the same time without reducing network performance. The protocol, to reach this objective, introduces five different enhancements in the RPL. The first seeks to prevent the network partition by allowing the “weaker” nodes, initially defined as leaves for running an MOP different from the root, to work as routers. The second enhancement allows non-storing nodes to send hop-by-hop DAOs, contrary to what happens in RPL, where non-storing nodes only send DAOs to the root. Thus, storing mode nodes should process and store DAO messages received from storing and non-storing nodes. The third and fourth enhancements suggest modifications in DAO message fields and adaptations in their processing for both storing and non-storing nodes. Thus, the DAO message can be fully and correctly understood by both modes, avoiding problems of ignoring fields. The fifth enhancement, defined as optional, modifies a flag inside of DAO message to inform whether the DODAG root uses storing mode or not. The information of this flag should be recorded in the routing table of storing mode nodes to optimize the construction of downward routes. Thus, DualMOP-RPL, with this set of improvements, enables the use of both storing and non-storing mode nodes without decreasing the network performance and optimizes the use of computational resource devices.

Considering that the solution designed to allow the use of different MOP on RPL is more focused on providing interoperability, Kim et al. [84] have proposed an improvement for enhancing the support of RPL to diverse traffic. In their work, the authors realized several experiments with the RPL protocol, identifying its performance decline in scenarios with downward traffic. Thus, a modification of RPL was proposed, named DT-RPL (Diverse Traffic-RPL), for providing a stable bidirectional communication among the network nodes. The approach explores the messages exchanged among the nodes to deliver link quality information during downward packet sending. In default RPL functioning, the nodes only update their link quality with an upward packet. Using DT-RPL, the downward packets carry link quality information in a specific reserved space inside of the IEEE 802.15.4 MAC header. Thus, the receiver of a downward packet computes the link quality and updates this information about its parent. This mechanism makes the routing table updating faster and avoids the constant triggering of global repair during the transmission of downward packets. Hence, DT-RPL makes possible the use of different traffic patterns and also contributes to the packet delivery ratio increasing and overhead control.

To overcome the problem existent in RPL related to dynamic load networks, Taghizadeh et al. [85] proposed a new routing protocol called Context-aware and Load-balancing RPL (CLRPL). The protocol aims to reduce the packet loss ratio and increase the network lifetime in LLN with heavy throughput and highly-variable traffic. The CLRPL approach is divided into three parts. The first, named CAOF (Context-aware Objective Function), is a new composed objective function that computes the rank of each node based on ETX, residual energy in both the node and its parent, and the rank of the parent. This information, which piggybacks on the DIO messages, is stored by the node in an array after being received. After calculating the rank of each DIO sender based on the stored information, CAOF uses an algorithm to sort the nodes by rank from best to worst. Thus, DIO with the best rank is broadcast first to avoid the thundering herd phenomenon [86]. The second part is the Context-Aware Routing Metric (CARF). CARF uses information about the status of the queue chain in the path, the rank of the node (computed using CAOF), and an index of network traffic dynamicity to obtain a value used in parent selection. As in CAOF, the information used by CARF is carried in the DIO. The third part is a parent selection mechanism that chooses the parent with the lower value computed by CARF. Whether or not the two candidate parents have the same CARF value, the mechanism selects the parent with the lower number of children nodes. The three parts are directly linked and exercise cooperative work. Thus, by considering the workload of the paths together with energy and link quality information, CLRPL can reduce energy consumption and improve the packet loss rate.

The routing solutions to support different traffic and MOP discussed above are summarized in Table 4.

### 4.5. Energy Efficiency and QoS Support

Reliability for data message sending and acceptable latency are requirements of all IoT applications. Due to power restrictions of various IoT devices, it is required that the packet sending task can be executed with the shortest energy expenditure possible. Therefore, a feasible QoS (Quality of Service) and reasonable energy consumption are considered as the most crucial features for all routing protocols for LLNs. Thus, in [87], Ancillotti et al. designed and developed a new cross-layer implementation of RPL, seeking to improve the data transmission reliability. The proposed solution, named RPLca+, is composed of two specialized libraries, the first for link quality estimation and the second for neighbor table management. The goal of the first library is to optimize the RPL operations through a hybrid link-monitoring framework to estimate the link quality with low overheads. Thus, the link quality library is composed of three methods of link quality estimation that are dynamically activated according to the information of the node and the characteristics of the link. The second library consists of a set of techniques for the management of neighbor tables. These techniques describe policies for insertion and replacement of entries in the RPL and IP tables seeking to manage better the neighbor’s information considering the memory limitation of the network nodes. Furthermore, the proposed implementation provides a synchronization mechanism between the RPL and IP neighbor tables to improve the consistency of the stored information. This synchronization is done in a unidirectional way in which the inserts and replacements realized in the RPL table are reflected in the IP table, but are not contrary. Finally, it is important to note that the proposal is interoperable with the default RPL implementation since it does not change the RPL control message fields.

A routing metric based on the transmission delay and remaining energy was proposed in [53], titled QoS_RPL (Quality of Service RPL). The authors used an Ant Colony Algorithm (ACO) [88] seeking to better fulfill the requirements of energetic efficiency and QoS in LLNs. During routing protocol functioning, the information about energy and delay are piggybacked on the control messages. This information is computed and updated by each node that receives a packet. Furthermore, the approach uses the pheromone (of ACO) as a metric in the process of route selection. The pheromone information is updated whenever a route is used to forward a data packet, realizing the process of path reinforcement. Thus, the most used paths tend to be better evaluated by the proposed approach. However, the authors also implemented negative reinforcement to prevent the use of suboptimal routes and allow the adoption of other possible, better paths. The QoS_RPL has presented the capacity of reducing the delay and consumed energy. Nonetheless, the packet delivery ratio has shown a slight reduction compared with RPL using the ETX metric.

A novel proposal for improving the network energy balancing seeking to maximize the lifetime of nodes was presented in [49]. The approach is based on RPL, and the authors focused on the creation of a routing solution that considers the estimation of energy consumption. Using a mechanism to measure the Expected Lifetime (ELT) of nodes and exploring multipaths, the proposed approach tries to avoid the use of bottlenecks (nodes with less energy) and equalizes the power consumption. Each node should compute its ELT based on the traffic expectation generated by itself and its children, the possible necessary retransmissions, the time during the transmissions, and the transmission power of its radio. The authors also suggested the use of a mechanism to limit the parental exchange. Thus, the quantity of a control message is reduced, contributing to fewer transmissions and energy depletion.

Qiu et al. proposed the ERGID (Emergency Response IoT based on Global Information Decision), a routing protocol that aims to provide both an efficient emergency response and reliable data transmission for IoT applications [89]. The proposal is based on two different mechanisms. The first, the Delay Iterative Method (DIM), classifies the nodes of a candidate route according to a global delay estimation. This mechanism is used to mitigate the problem of ignoring a valid path. Furthermore, DIM seeks to ensure real-time communication for emergency response applications and performs periodical updates in the routing table. The second mechanism, called Residual Energy Probability Choice (REPC), enables the use of residual energy information during the process of next node selection to forward a message. Thus, through the composition of these mechanisms, ERGID gives a low end-to-end delay (provided by DIM) and an efficient energy consumption distribution (supplied by REPC).

A Neighbor Disjoint Multipath scheme attached to the LOADng routing protocol (LOADng + NDM) was introduced in [90]. The proposed scheme seeks to reduce the problems caused by local node failures and a lousy channel condition in specific areas. During its functioning, LOADng + NDM first creates a primary path between the source and destination nodes, which is frequently the shortest route. In sequence, the scheme tries to build a set of backup paths. These alternative routes need to attend to some requirements, such as: none of its nodes can compose the primary path, except to source and destination nodes; and none of its nodes can be a neighbor of the nodes that form the primary path, except to source and destination nodes. Using this approach and considering applications with P2P traffic, LOADng + NDM can overcome the default LOADng regarding throughput and latency. It is also important to emphasize that the proposed neighbor disjoint multipath scheme can be used with different routing protocols, including the well-known RPL.

A framework for co-operating with routing protocols and providing QoS and energy efficiency for IoT networks was presented in [91]. Considering IoT scenarios that merge RFID and IEEE 802.15.4 devices, the proposed solution presents two mechanisms to improve the performance of both the routing protocol and the RFID tag-reading process. The first mechanism, named the Fuzzy Q-Algorithm (FQA), is a tag anti-collision protocol especially developed for IoT scenarios in which the tag reading process is realized for LLN devices equipped with RFID readers. FQA uses a fuzzy system to adjust the *Q* value [92] dynamically according to the tags’ density and improve the reading process, making it faster and reliable. The second mechanism, called the Fuzzy System-Based Route Classifier (FSBRC), is a fuzzy system designed for joining four routing metrics (node energy, hop count, tags’ density, and LQI) and defines a unique value that determines the route quality. The FSBRC uses the routing protocol control messages for transporting the metrics, which after being received, are used in the path quality computation. The nodes should select the path with higher route quality value to forward the data packets. Thus, the framework integrates both mechanisms to enhance the performance of IoT networks with heterogeneous devices and different wireless technologies. In the paper, the authors applied the proposed approach in the Directed Diffusion (DD) [93] routing protocol. However, they emphasized that the solution can be used with different protocols such as RPL and LOADng.

Sasidharan and Jacob [94] have proposed a new routing metric and a multipath forwarding mechanism for LOADng aiming to improve the network energy efficiency, load balancing, and data message exchange reliability. The introduced composite routing metric, named LRRE, is based on three primary metrics: hop count, residual energy, and live routes. LRRE uses different parameters to allow the adjustment of the influence of each primary metric in the route value computation. Thus, α, β, and γ are used to tune the weight of residual energy, live routes, and hop count, respectively. Each weight is multiplied by its corresponding primary metric, and in the sequence, all the values are summed to obtain the LRRE value. The computed value is carried inside of default LOADng control messages (RREQ and RREP), not requiring additional fields. Hence, the cost of a path is the sum of the received LRRE value inside the message plus the value computed by the node. In addition to LRRE, the authors have also proposed adaption for LOADng that allows a node to create more than one route to the same destination. Thus, in the proposed functioning adaption, when an RREQ message is received, the node checks if the number of existing paths to the RREQ originator is lower than three. If true, a new route is added to the routing table. Otherwise, if the route indicated by the received RREQ is better than the one already existent, the new route should replace it. Finally, the authors’ proposal also included a new Weighed Forwarding (WF) mechanism to allow the routing of data messages through the paths with the best LRRE values. The proposed set of improvements allows LOADng to construct multiple paths to a unique destination and provide load balancing and reliability in the message exchange process. However, this approach can increase memory usage and affect the network scalability, restricting the proposal adoption in large-scale IoT applications.

Due to implementation costs, it is expected that in several IoT scenarios, not all devices can have the capacity for direct communication with the Internet. In this case, the nodes without this capacity can use Internet-Connected (IC) devices as a gateway (or bridge) to communicate with external services and applications. Based on this specific heterogeneous IoT scenario, Sobral et al. [95] have proposed LOADng-IoT. The proposal introduces a new route discovery mechanism for the nodes to find IC nodes automatically without the need for a predefined gateway configuration. Thus, a node that wants to find a device to use as its gateway (i.e., an IC device) uses an RREQ-IoT message. The RREQ-IoT is broadcast and forwarded as a normal RREQ from default LOADng. However, the RREQ-IoT does not have a specific destination and can be replied by any device with an Internet connection in the network. When an IC node receives the RREQ-IoT, an RREP-IoT message is generated and sent to the request originator. The RREP-IoT is forwarded through the reverse path created by the RREQ-IoT until its destination. Each intermediate node that receives the RREP-IoT should add the message originator as a gateway to the Internet. Thus, in case there is a future need to send the message to the Internet, a path to an IC device is already known. LOADng-IoT also introduces a route cache mechanism to reduce the overhead of the route discovery process and a new error message used to inform when an IC node has lost its Internet connection. Thus, the proposal allows LOADng to better attend to the requirements of the QoS and energy efficiency needed by several IoT applications.

Table 5 shows a summary of studied solutions for energy efficiency and QoS support in IoT/6LoWPAN networks.

### 4.6. RPL Objective Functions

The RPL protocol uses objective functions to determine the network topology and the process of preferred parent selection. In its RFC document, RPL does not mandate the use of a specific OF and suggests that this choice should be made based on the application requirements. However, IETF has defined two initial OFs, OF0 and MRHOF, which can attend to simple routing requirements. Nonetheless, these OFs can present some limitations, e.g., they do not consider energy information during route selection. Thus, several works have proposed alternative OFs for RPL considering different routing metrics.

The Energy-Aware Objective Function (EAOF) was introduced in [96]. EAOF seeks to increase the network lifetime and reliability of Biomedical Wireless Sensor Networks (BWSN). According to the authors, a BWSN should be able to work without human intervention for an extended period to further require reliable levels of QoS and efficient energy consumption. Thus, EAOF uses ETX and Remaining Energy (RE) to compute the best path to the root node aiming to satisfy the requirements of BWSN. EAOF performs the best parent selection in three steps and using two configurable parameters: MAX_ETX and MIN_ENER. In the first step, the proposed OF uses MAX_ETX as a threshold to define the maximum value that a potential parent should have to be considered in the next step. Thus, a node only can be considered as a candidate to be the best parent if it has an ETX value to the sink lower than MAX_ETX. In the next step, EAOF compares the rank of the candidate with the rank of the node. In the last step, the node with the highest RE is selected. EAOF introduces some hysteresis through the MIN_ENER parameter. Hence, a potential node only should be selected as the new preferred parent if the difference between its RE and the RE of the current parent is higher than MIN_ENER. This way, EAOF seeks to select the preferred parent with lower ETX and higher RE.

Some approaches do not propose a completely new OF, but improve how the rank of the nodes is computed, introducing a new routing metric. In [97], the authors proposed an additive composite metric called the Lifetime and Latency Aggregatable Metric (L${}^{2}$AM). L${}^{2}$AM aims to provide balanced energy consumption considering the reliability of data transmission along the path. To this end, the proposed routing metric merges a link reliability metric (i.e., ETX) with a new energy consumption metric termed Fully Simplified Exponential Lifetime Cost (FSELC). FSELC represents the power cost that each node needs to pay to send a message. During the RPL functioning, the nodes use DIO messages both for transporting information about the metrics and for informing the L${}^{2}$AM value computed for each neighbor. The L${}^{2}$AM value must be summed along the path to obtain the overall route cost. When a node needs to send a packet to the root, it should select the path with the lowest summation of L${}^{2}$AM. Thus, the proposed metric allows the node to use routes that are reliable and energy efficient.

A fuzzy-based OF was presented in [74,98]. The proposed OF-FL (Objective Function Fuzzy Logic) aims to create a robust OF that can fulfill different routing requirements through consideration of several network metrics. In the work, the authors highlighted that only one, or a simple combination of, routing metrics cannot be sufficient to create a reliable path between a sender and a receiver. Thus, OF-FL uses a fuzzy system to combine four primary routing metrics (end-to-end delay, hop count, link quality, and node energy) and obtain a value that indicates the quality of a neighbor. As any OF, the information used to compute the rank of nodes piggybacks onto DIO messages. Hence, the nodes that receive a DIO execute the fuzzy system for computing the rank of the message sender neighbor. After, looking for the set of neighbors and its respective qualities, the OF-FL selects the best preferred parent based on the application requirements. Thus, OF-FL permits RPL, considering different network aspects during the route construction phase.

Another fuzzy-based OF was introduced in [99]. The proposal sought to merge different routing metrics to optimize QoS and energy consumption. The FUZZYOF combines three routing metrics, which are the delay, ETX, and energy, to obtain a fourth value termed quality that represents the path cost. Delay is the average time it takes for a packet to reach its destination. ETX, as already introduced, is the expected number of transmissions for a message to be delivered successfully. The energy metric expresses the lowest energy level among the nodes that compose a path. Different from the previously-presented OF-FL, FUZZY OF obtains the quality of a route using two fuzzification processes. The first considers the two metrics: delay and ETX. As a result of the first process, an output called QoS is produced. This QoS output, together with the energy metric, are the two inputs of the second fuzzification process, which provides the path quality value. Notice that the result of both fuzzification processes is a maximizable value. Thus, the node should select, as its preferred parent, the neighbor candidate with the highest quality value. In FUZZY OF, the rank value of a node is obtained through the sum of the rank of its preferred parent with respective computed quality. Thus, the proposed OF allows RPL to construct a routing topology considering both QoS and energy aspects.

Araujo et al. [100] also adopted fuzzy systems to design a new set of context-aware objective functions for RPL. The authors aimed to provide a solution that can dynamically adjust its functioning to better attend to the requirements of the applications. Thus, they defined four Delivery Quality- and Context-Aware Objective Functions (DQCA-OF) considering the metrics of ETX, consumed energy, and the number of hops. A priority level was also defined for each metric used in DQCA-OFs. As an example, DQCA-OF1 was designed with the additive combination of the routing metrics ETX and number of hops. Each one of these metrics has a priority level that varies among high (1), medium (3), and low (5). Thus, the DQCA-OF proposal stored the routing needs of applications inside a database to adjust the priority level of metrics according to the requirements of each scenario. The authors also proposed a fuzzy system to combine the metrics of DQCA-OFs, alternatively. Thus, for each DQCA-OF exists a DQCA-OF (FL) that merges the routing metrics using the proposed fuzzy system to classify the routes. Therefore, the authors created a set of eight OFs that can be dynamically chosen according to the IoT application requirements for improving energy usage and QoS.

An objective function, developed for a specific implementation of RPL for Agricultural LLNs (A-LLNs), is presented in [101]. The Scalable Context-Aware Objective Function (SCAOF) introduces context-aware features in RPL to fulfill the QoS requirements of A-LLNs. The proposed OF combines Link Color Object (LCO) metrics and an additive metric composed of ETX and RE (remaining energy). The LCO is used to propagate information about the links, such as workload, hardware, and availability. The RE metric is an additive metric that represents the average remaining energy of the nodes that compose a path. All this information is obtained locally by the nodes or exchanged using DIO messages. To select its preferred parent, a sensor node seeks its parents with the appropriated LCO, and afterwards, it chooses the one with the lowest second metric (composed of ETX and RE). Next, the node should compute its rank based on ETX and RE and subsequently forward it to its neighbors. Hence, SCAOF provides the combined use of several routing metrics to fulfill the requirements of A-LLNs, mainly QoS and energy efficiency.

Gozuacik et al. [102] proposed the Parent-Aware Objective Function (PAOF), an objective function that aims to offer network load balancing for LLNs. PAOF uses both ETX and parent count to perform the rank computing and preferred parent selection. The parent count metric represents the number of potential preferred parents of the node. These two metrics are combined in a lexical way. In the comparison between two candidate nodes, PAOF first verifies the ETX modular difference between them. Whether it is smaller than MinHopRankIncrease or not, the node should select the best parent to be the candidate with the lowest parent count. MinHopRankIncrease is a default variable value of RPL that defines the minimum value increased in the rank for each parent of the node. Thus, although PAOF considers two routing metrics, the primary decision is based on the ETX, the second metric being used just in case of a significant difference among ETX values of candidates nodes.

The main features of the OFs studied during this subsection are shown in Table 6.

## 5. Discussion, Lessons Learned, and Open Issues

The last section presented the most relevant and recent approaches for improving the performance of routing layers in IoT/LLN networks. At the end of each subsection, comparison tables summarizing the most relevant characteristics (strengths and weaknesses) of the described protocols were included. Thus, it was possible to observe several relevant aspects of the current approaches.

The studied routing solutions for LLNs are, in general, based on the RPL routing protocol. This is justified due to RPL being an already consolidated and well-known solution. Furthermore, RPL, can initially better attend to the routing requirements presented by IETF when compared to other standard routing protocols. Furthermore, some approaches use solutions based on the combination of RPL with other already existent proposals. These approaches seek to solve the RPL drawbacks with the benefits of other previously-known proposals. Although these approaches can improve the standard RPL, the final algorithm tends to be long and complicated, requiring a higher storage and processing capacity of the nodes. According to the results presented in each work, the majority of the studied solutions can improve the packet delivery rate (or decrease the packet loss) and reduce energy consumption. Thus, it is clear that the RPL has various problems with packet delivery reliability and energy efficiency.

The broad adoption of RPL makes the most recent approaches attempt to overcome the existing limitations in the IETF routing standard. The costly support to P2P communication, provided by RPL, motivated the creation of new approaches such as P2P-RPL and AODV-RPL. These are, as well as LOADng, inspired by AODV. However, the inclusion of a reactive mechanism into RPL can increase the control message overhead and contribute to the increase of energy consumption. As alternatives, other solutions for P2P support improvement are based on geographic routing. These proposals tend to reduce the control message overhead and provide scalability, a critical requirement for several IoT applications. However, the necessity of location-aware nodes (e.g., equipped with GPS) can make its adoption very expensive.

Another feature defined continuously as a requirement of LLN applications is the support to multicast transmission. Industrial, home automation, and urban and building applications (as shown in Section 2) can present the necessity of realizing transmission for pre-defined groups of nodes. MPL, SMRF, ESMRF, and most recently, BMRF, are the main approaches to allow devices with limited computational resources to perform multicast transmission with high reliability and low energy consumption. However, these solutions tend to execute coordinated floods of messages or present cross-layer approaches. Such characteristics make their adoption costly regarding packet overhead and, in the case of cross-layer approaches, cause the dependency of using a specific MAC protocol [103] and the increasing end-to-end latency.

A considerable part of the current and future IoT/LLN applications admits the use of mobile devices. However, when neglected, the topology changes provoked by the movement of the nodes can cause strong adverse effects in network performance. Thus, mobile nodes support emerges as a requirement for all groups of IoT applications. As the current version of RPL does not present robust support for mobility, several works have been introduced to overcome this limitation. The approaches are very diverse and range from the use of merging RPL with another already existent solution to the adoption of movement prediction mechanisms. Although these approaches can improve mobility support when compared with RPL, they usually increase end-to-end latency and require extensions in the routing tables or increase the control messages’ length.

The variety of IoT applications is unbounded, as well as its requirements of throughput and traffic flow. The main drawback of LOADng is the weak support of MP2P traffic. To surpass this limitation, LOADng-CTP introduces new features to allow protocol to work based on collection tree routing. In contrast, RPL presents four different kinds of MOP that can be adopted, one at a time, according to the traffic requirements of the application and hardware of the devices. However, some applications could take advantage of using more than one MOP to improve their operation or to allow new functionalities. Thus, a dual-MOP solution was proposed to attend to this necessity. Furthermore, IoT applications can change their traffic pattern and load according to specific events or times. These requirements have motivated the creation of DT-RPL and CLRPL. These solutions can offer a robust self-adjustment capacity to attend to applications’ requirements. In contrast, the protocol complexity, size of control messages, and latency can be widely increased. In the same way, a higher hardware capacity of devices is required.

Energy efficiency and QoS are requirements of the majority of IoT applications. Thus, LLN routing solutions should provide reliability for packet delivery with low latency and efficient energy consumption. The current proposals try to meet these necessities by using cross-layer approaches, multipath solutions, and computational intelligence algorithms, among others. The literature also presents solutions based on protocols different from RPL, such as ERGID, LOADng + NDM, LRRE + WF, and LOADng-IoT. Usually, OFs for RPL are proposed to attend to these same requirements. The studied OFs can improve the performance of RPL when compared with the standard OFs (e.g., OF0 and MRHOF). The OFs that consider a higher number of routing metrics tend to have better results. However, the implementation of a composed metric may not be trivial. Some solutions use fuzzy systems to perform the composition of routing metrics as an alternative to the conventional lexical and additive methods. Although fuzzy system-based OFs allow domain experts to be able to map their experience and decision-making processes to define the quality of a path, their implementation tends to be complex and requires more computational resources compared with traditional composition methods.

The presented discussion about the studied routing solutions has identified that the main constraints of the current routing solutions are the increment of algorithm complexity, the extension of message length, the increment of the use of control messages, and the implementation overheads. Furthermore, the majority of current solutions are evaluated employing computational simulations. Although simulations allow a higher variation of scenarios and number of nodes, their results may not represent the real performance of the approach. Beyond that, the current approaches designed for specific applications do not consider the routing requirements presented by IETF and RoLL for each application group. Although these considerations are not mandatory, it is important to note that they can represent the right direction towards a satisfactory functioning of a routing solution.

The development of routing solutions that can provide a stable service for the application layer of the IoT network is not an easy task. The different application requirements demand an acceptable trade-off among the various aspects of the network. Thus, some open issues and guidelines suggested for consideration in the creation of new approaches are as follows:

- Considering devices’ heterogeneity: The current routing solutions are, usually, designed and evaluated considering just one or two kinds of devices. However, one of the first assumptions of IoT applications is the interoperability among heterogeneous devices. Different hardware configurations can affect the performance of routing protocols and the rate of message processing. Thus, it is critical that the development of a routing solution for IoT be conducted considering the diversity of hardware devices.
- Seek a satisfactory trade-off among the network aspects: As mentioned in the course of this work, the requirements of the 6LoWPAN routing protocol can vary according to the applications. Some applications can demand reliability, while others want energetic efficiency. However, the requirements of applications are frequently concurrent among them. As an example, the improvement of the network load balancing can affect the end-to-end delay negatively. Thus, routing solutions must seek a satisfactory trade-off among the concurrent requirement of the applications.
- Robust solutions with low complexity: The approaches currently presented seek to cover drawbacks, improve the weaknesses of an existent solution, or attend to as many requirements as possible. However, the design of an enhanced or “perfect” solution can generate unexpected results. With the increment of algorithm complexity, devices can be overloaded, causing a decrement to the network performance. With this, the approach needs to be robust to consider all the relevant requirements in the scope of the applications and, at the same time, be simplistic and light for execution in devices with low computational power.
- Consider the IETF’s recommendations: Although the consideration of the IETF specifications is not mandatory, several efforts were demanded to define the routing requirements for the different types of IoT/6LoWPAN applications. When considering the study approaches in the overview of this work, the routing solutions developed for specific applications do not consider IETF’s recommendations. The consideration of IETF’s guidelines can allow the proposal for a satisfactory behavior, high acceptability, and the future standardization of the solution.
- Interoperability with base protocols: The majority of studied routing solutions are based on already existent routing protocols. Thus, they use almost all the structure of the base protocols as RPL and AODV. However, a small number of current approaches provide support and interoperability with the protocol on which they are based. This gap makes the adoption of these new solutions difficult in an already functioning IoT network, because it would require updating the firmware of the nodes. Thus, it is recommended that new solutions can interact with the base protocols, allowing the coexistence of both in the same network. A way to attend to this requirement is by avoiding significant changes in the fields and processing the mode of control messages.
- Compatibility of existent OSs and boards: The current state-of-the-art presents a large set of boards (Arduino, WSN430 open node, M3 open node, TelosB, Zolertia Z1) that are constantly used in IoT scenarios. In recent years, several Operation Systems (OS) [104,105] were especially developed for the IoT context, e.g., Contiki OS [70] and RIOT OS [106]. It is desired that the new routing solution can be executed by the most recent adopted boards and OS for IoT. To this end, the proposal should be exhaustively tested through emulation, simulation, and real testbeds (although this can be expensive). The RENODE^™^ [107] can be helpful to reach these features.
- Make available and reproducible proposals: Although it is possible to find several routing proposals in the literature, the number of these that can be entirely reconstructed and studied is low. Commonly, the routing solution proposals are superficially described, and many details are suppressed. Thus, it is difficult to perform an accurate reproduction of the presented approaches. Providing flowcharts, algorithms, message structures, and mathematical equations helps the proposal to be better understood and makes it implementable. Because of this, the approach can be considered for comparison with another proposal or utilized for real IoT application. It is also essential to show a full description of the testbed or simulation parameters to allow a fair comparison among the solutions. It is desirable that future approaches can make public their implementations, simulation scripts, and trace logs, whenever possible.
- Self-adaption to the application context: Some applications can present different needs according to the time or type of data message. As an example, at a given time, an application can require low latency and may continue to need high reliability or lower energy consumption. Thus, routing solutions should self-adapt to the temporary context of applications. This feature can be obtained with the creation of cross-layer solutions in which the application layer indicates their particularities to the routing layer. However, it is important to highlight that some application can never require this characteristic. In this case, they can adopt a straightforward approach.

## 6. Conclusions

The creation of the 6LoWPAN working group by IETF allowed the development of a solution to provide the use of IPv6 over low power devices. This contribution boosted the emergence of IoT applications. However, since the definition of LLNs, the routing in the networks with low power devices is considered a significant challenge. Seeing the importance of studying appropriate routing solutions, IETF created the RoLL working group. Initial conclusions of a study developed by the new working group showed that the existent IETF standard routing protocols were unable to provide the routing requirement of LLNs. Thus, RoLL began the development of a new routing solution considering the specificities identified by the group for different LLN applications.

Although the RoLL working group has defined RPL as the standard routing protocol for LLNs, several works have shown that the solution presents severe limitations and drawbacks. Thus, various new solutions studied in the course of this document have emerged in recent years trying to mitigate the existent routing issues. The currently-proposed enhancements are designed, in general, to solve problems related to P2P and mobility support, multicast data forwarding, energy efficiency, and QoS improvement. However, it is important to note that these new routing solutions are for the IoT/LLN network, as they are not limited to RPL enhancements. LOADng and its advances have emerged as a potential solution, especially for P2P communication, though it is much less studied than RPL in the recent literature.

The comprehensive analyses and discussions about the studied routing strategies have allowed the identification of the evolution of state-of-the-art solutions. However, it is also possible to point out various limitations of current proposals, which shows that they are still unable to fulfill the IoT routing requirements feasibly. Finally, several research directions and guidelines were presented for consideration in the design of new proposals.

## Figures and Tables

**Table 1:** Comparison among studied approaches for P2P communication support. GOAFR, Greedy Other Adaptive Face Routing.

Proposal	Base Protocol	Objectives	Description	Strengths	Weaknesses	Target Applications
P2P-RPL [61]	RPL	- Improve P2P support of RPL	- Creates new P2P routes on demand as an alternative to P2P routes built by RPL	- Offers the creation of alternative P2P routes to fulfill the application routing requirements - Avoids the use of a root node to forward P2P messages	- Floods the network with control packets that can increase the overhead and energy consumption	- Applications that require P2P traffic
GeoRank [63]	RPL and GOAFR	- Improve P2P support and reduce the amount of control messages	- Combines RPL with a geographic routing approach to reduce control message in P2P communication	- Avoids the use of DAO messages - Improves scalability, reducing the memory usage	- Changes the default RPL control messages - Requires static nodes or equipped with GPS	- Smart street lighting systems (as an example)
ER-RPL [65]	RPL	- Provide an energy-efficient and reliable P2P communication	- Performs a region-based P2P route discovery to save energy and create direct paths	- Avoids the P2P control message flooding by the whole network, hence producing a considerable energy savings - Improves the P2P packet delivery ratio	- Requires some location-aware nodes (e.g., with GPS) - Complex approach - Introduces new control messages in addition to default RPL messages	- Applications that require P2P traffic
AODV-RPL [66]	RPL and AODV	- Improves the support for the P2P traffic pattern of RPL	- Creates paired DODAGs for P2P message exchange through asymmetric routes	- Considers the bidirectional link condition during route discovery - Can reduce the size of control messages	- The current version is still in draft form - Has not presented performance results yet	- Applications that require P2P traffic

**Table 2:** Comparison among studied approaches for multicast communication support.

Proposal	Base Protocol	Objectives	Description	Strengths	Weaknesses	Target Applications
MPL [67]	-	- Provide multicast data forwarding in LLNs	- Uses the flooding mechanism governed by the trickle algorithm	- Promotes a high packet delivery ratio due to buffer messages and retransmits it locally when necessary	- Can provoke a communication overhead - Low storing capacity of devices can limit the buffer size, causing a performance reduction	- Applications that require multicast transmission
SMRF [68]	RPL	- Enhance the multicast data forwarding provided by RPL	- Cross-layer approach that optimizes the ContikiMAC for multicast transmission	- Reduces the energy consumption occasioned by multiple unicast transmission in multicast RPL - Avoids processing of duplicated multicast packets	- Only allows downward multicast transmission - Can provoke high end-to-end latency	- Applications that require multicast transmission
ESMRF [71]	RPL and SMRF	- Permit both upward and downward multicast data forwarding in SMRF	- Encapsulates multicast messages and sends them to the root node to perform the forwarding	- Solves the gap of SMRF by allowing upward multicast traffic	- Oversees the communication by using the root node to forward all multicast traffic - Can cause high end-to-end latency	- Applications that require multicast transmission
BMRF [72]	RPL and SMRF	- Enhances both upward and downward multicast data forwarding in SMRF	- Merges the features of RPL and SMRF to offer different transmission modes used according to the needs	- Can reduce the number of radio transmissions and energy consumption - Increase the packet delivery ratio	- Slightly higher memory consumption - Increases the end-to-end latency - Wrong parameter adjustment can provoke low performance	- Applications that require multicast transmission

**Table 3:** Comparison among the studied approaches for mobile nodes’ support.

Proposal	Base Protocol	Objectives	Description	Strengths	Weaknesses	Target Applications
Co-RPL [73,74]	RPL	- Improve the mobility support of RPL	- Routing solution based on the corona mechanism	- Presents an alternative mechanism to path recovery - Reduces the packet loss ratio, the average energy consumption, and the end-to-end delay	- Requires changes in the default RPL messages - Requires extension of the routing table	- General mobile wireless sensor network applications
mRPL [75]	RPL and smart-Hop	- Provide a fast and reliable mobility support in RPL	- Integrates the smart-Hop mechanism with RPL	- Presents a mechanism for collision and loop avoidance - Interoperable with default RPL - Reduces packet loss rate and delay - Source code available for Contiki OS	- Short increment in the control messages’ length - Increases the number of exchanged control messages	- Wireless clinical monitoring applications
Mod-RPL [54]	RPL	- Adjust RPL for hybrid networks (mobile and static nodes)	- Modification of RPL to limit the operation of mobile nodes	- Reduces the use of control messages - Interoperable with default RPL	- Forbids the use of mobile nodes as routers - Only slow mobile nodes are considered	- Healthcare and medical applications
EKF-RPL [78]	RPL	- Improves mobility support of RPL and reduces the signaling overhead of mobile nodes	- Uses the extended Kalman filter to predict the movement of mobile nodes and to reduce the parent changes	- Reduces the control message overhead and energy consumption - Mobile nodes select the parent that can offer a higher linkage time	- Can increase the end-to-end latency - Does not consider important information as energy and link quality in the parent selection	- Application with mobile nodes
BRPL [79]	RPL and backpressure routing	- Enhances RPL to support mobility and dynamic traffic load	- Combines RPL with backpressure routing concepts to distribute the network resources adaptively	- Provides a considerable packet loss reduction - Can coexist in a network already running default RPL	- Increases the end-to-end delay significantly	- Application with mobile nodes and variable throughput
EMA-RPL [81]	RPL	- Increases the lifetime of mobile devices and improves network connectivity	- Monitors RSSI to predict the movement of the mobile nodes and promote the change of the preferred parent	- Use of power and computational resources of mobile nodes is reduced - Does not require the use of additional hardware for mobile detection (e.g., GPS)	- Mobile nodes cannot route packets from other nodes - Requires several additional fields on standard RPL control messages	- Healthcare applications

**Table 4:** Comparison among studied approaches for different traffic and MOP support. CTP, Collection Tree Extension.

Proposal	Base Protocol	Objectives	Description	Strengths	Weaknesses	Target Applications
LOADng-CTP [82]	LOADng	- Permit LOADng to efficiently support MP2P traffic	- Introduces proactive features in LOADng for creating a bidirectional routing tree	- Significantly reduces the overhead and latency of LOADng in MP2P traffic - Easy implementation over LOADng core	- Introduces extra fields on default LOADng messages and a new HELLO message - Implementation requires new data structures that can increase the memory usage	- Not specified
DualMOP-RPL [83]	RPL	- Enable the use of both storing and non-storing MOP in a single RPL network	- Introduces a set of modification in RPL control messages and enhances its processing mode	- Solves interoperability problems existent between the two MOPs	- Increases the complexity of control messages’ processing - Modifies the structure of the standard RPL control message	- Applications with heterogeneous devices
DT-RPL [84]	RPL	- Improves the reliability of RPL for different traffic patterns	- Allows a bidirectional measurement of link quality during the message exchange	- Improves the RPL performance during downward-centric communication - Does not require significant changes in the default RPL	- Does not consider energy information for load balancing - Can present limitations in scenarios with many nodes	- Not specified
CLRPL [85]	RPL	- Enhances support to heavy and highly dynamic load LLNs	- Creates new mechanisms to consider information about node workload, energy, and link quality in the parent selection process	- Uses relevant information in the parent selection - Reduces the changes of the preferred parent - Reduces energy consumption and increases PDR	- Requires an extra memory consumption and message sorting - Can increase the end-to-end delay	- Application with heavy and dynamic traffic load

**Table 5:** Comparison among studied approaches for energy efficiency and QoS support.

Proposal	Base Protocol	Objectives	Description	Strengths	Weaknesses	Target Applications
RPLca+ [87]	RPL	- Improves the reliability of data delivery in RPL	- Cross-layer approach for link quality estimation and routing table management	- Provides a dynamic link quality estimator - Provides the policies for the management of routing tables - Improves the packet delivery rate	- Presents implementation overhead - Increases the energy consumption	- Advanced metering infrastructure
QoS_RPL [53]	RPL	- Improves energy efficiency and QoS in LLNs	- Composite routing metric based on delay, remaining energy, and pheromone (ACO)	- Reduces the delay and energy consumption	- Decreases the packet delivery ratio - Can provoke a disturbance in the load balancing - Increases the control message overhead	- Not specified
multiELT-RPL [49]	RPL	- Maximizes the network lifetime and creates an energy balanced topology	- Computes the expected node lifetime and uses a multipath RPL modification	- Increases the node lifetime - Prevents the early necessity of routing topology reconfiguration	- Increases the memory usage - Modifies the structure of default RPL control messages	- Not specified
ERGID [89]	SPEED	- Provides low delay and load balancing for emergency response applications	- Uses the estimation of global delay of nodes and information about the energy consumption	- Chooses routes based on global information - Reduces the average delay and packet loss rate without increasing energy consumption	- Uses a high number of control messages to maintain delay information updated - Requires frequent calculations and routing table update	- Emergency response applications
LOADng+NDM [90]	LOADng	- Reduces problems caused by local failures in the nodes or radio interferences	- Creates a set of alternative neighbor disjoint routes	- The main scheme can be adapted for different routing protocols - Avoids the usage of network regions with local problems	- Does not consider any information about energy consumption or link quality in the route selection - Increases the use of control messages and the complexity of the protocol	- Not specified
FQA + FSBRC [91]	DD	- Improves QoS and energy efficiency in IoT networks composed by RFID and LLN devices	- Designs a new tag-reading protocol integrated with a fuzzy system-based route classifier that uses four routing metrics to define the path quality	- Considers IoT devices equipped with RFID readers - Allows the consideration of RFID tags’ density in the node area during the route selection process	- The approach is based on fuzzy systems that can be complex for IoT devices - The improved reading tags’ algorithms requires changes in the standard protocol	- Applications that englobe RFID and LLN devices
LRRE + WF [94]	LOADng	- Improves the network lifetime, load balancing, and reliability	- Introduces a new composite routing metric and a new multipath route discovery and forwarding mechanism for LOADng	- Permits LOADng to construct multiple paths between source and destination nodes - Provides load balancing and improves network energy efficiency	- Proposed multipath adaption can increase memory usage and affect the network scalability - Bad adjusting of the parameters of the proposed routing metric can decrease the network performance	- Machine-to-machine communication applications
LOADng-IoT [95]	LOADng	- Allows LOADng to better discover and maintain routes in heterogeneous networks	- Introduces a new route discovery mechanism dedicated to creating paths to Internet-connected nodes	- Permits the nodes to find gateways to the Internet without a previous configuration - Presents a solution to reduce the overhead generated during the route discovery	- Requires the insertion of an extra field on the default LOADng control messages - Route cache mechanism can increase the memory usage	- Applications where nodes have different Internet connection capacities

**Table 6:** Comparison among studied RPL objective functions. L${}^{2}$AM, Lifetime and Latency Aggregatable Metric.

Proposal	Base Protocol	Objectives	Description	Strengths	Weaknesses	Target Applications
EAOF [96]	RPL	- Provides energy efficiency and balance	- Lexical composite OF based on ETX and remaining energy	- Increases the network lifetime - Easy implementation	- Packet delivery rate is decremented	- Biomedical WSN applications
L${}^{2}$AM [97]	RPL	- Performs energy consumption balancing and improves reliability	- Additive composite OF based on energy and ETX	- Compatible with default RPL - Easy implementation - Improves network lifetime	- Packet delivery ratio is not studied	- Not specified
OF-FL [74,98]	RPL	- Provides a configurable routing decision with a fuzzy system	- OF based on fuzzy systems that mix end-to-end delay, ETX, node energy, and hop count	- Routing decisions are made considering different network aspects - Presents improvement in the end-to-end delay, network lifetime, and packet loss ratio	- Implementation of a fuzzy system can extend the memory usage - The definition of fuzzy parameters is not trivial and can strongly affect the network performance	- Not specified
FUZZY OF [99]	RPL	- Improves the QoS of RPL using fuzzy systems	- OF based on fuzzy systems that mix energy, delay, and ETX	- Reduces the packet loss ratio - Reduces the end-to-end delay	- Implementation of a fuzzy system can extend the memory usage - The definition of fuzzy parameters is not trivial and can strongly affect the network performance	- Not specified
DQCA-OF [100]	RPL	- Attends to the routing requirements of various IoT applications	- Set of OFs based on ETX, number of hops, and energy consumption combined using fuzzy systems	- Can change its features in running time to attend to the routing requirements - Improves network QoS	- Implementation of a fuzzy system can extend the memory usage - Requires previous knowledge of applications	- Not specified
SCAOF [101]	RPL	- Enhances the applicability of RPL in agricultural LLN, providing QoS	- Additive and lexical composite OF based on energy, link color, and ETX	- Performance evaluation realized in a real testbed - Allows the extension of network lifetime - Increases network reliability and efficiency	- Complex approach - Requires an extended version of RPL	- Agricultural LLNs
PAOF [102]	RPL	- Provides load balancing	- Lexical composite OF based on parent number and ETX	- Distribution of work load of nodes - Increases the network lifetime	- Does not consider node energy information	- Not specified

## References

[1] Al-Fuqaha A., Guizani M., Mohammadi M., Aledhari M., Ayyash M. (2015). Internet of Things: A Survey on Enabling Technologies, Protocols, and Applications. IEEE Commun. Surv. Tutor..

[2] Atzori L., Iera A., Morabito G. (2010). The internet of things: A survey. Comput. Netw..

[3] Council N. (2008). Disruptive Civil Technologies: Six Technologies with Potential Impacts on Us Interests Out to 2025.

[4] Gluhak A., Krco S., Nati M., Pfisterer D., Mitton N., Razafindralambo T. (2011). A survey on facilities for experimental internet of things research. IEEE Commun. Mag..

[5] Vermesan O., Friess P. (2013). Internet of Things: Converging Technologies for Smart Environments and Integrated Ecosystems.

[6] Díaz M., Martín C., Rubio B. (2016). State-of-the-art, challenges, and open issues in the integration of Internet of things and cloud computing. J. Netw. Comput. Appl..

[7] Domingo M.C. (2012). An overview of the Internet of Things for people with disabilities. J. Netw. Comput. Appl..

[8] Perera C., Zaslavsky A., Christen P., Georgakopoulos D. (2014). Context aware computing for the internet of things: A survey. IEEE Commun. Surv. Tutor..

[9] Rodrigues J.J., Neves P.A. (2010). A survey on IP-based wireless sensor network solutions. Int. J. Commun. Syst..

[10] Montenegro G., Schumacher C., Kushalnagar N. (2007). IPv6 over Low-Power Wireless Personal Area Networks (6LoWPANs): Overview, Assumptions, Problem Statement, and Goals.

[11] Montenegro G., Hui J., Culler D., Kushalnagar N. (2007). Transmission of IPv6 Packets over IEEE 802.15.4 Networks.

[12] Thubert P., Hui J. (2011). Compression Format for IPv6 Datagrams over IEEE 802.15.4-Based Networks.

[13] Kim E., Kaspar D., Vasseur J. (2012). Design and Application Spaces for IPv6 over Low-Power Wireless Personal Area Networks (6LoWPANs).

[14] Kim E., Kaspar D., Gomez C., Bormann C. (2012). Problem Statement and Requirements for IPv6 over Low-Power Wireless Personal Area Network (6LoWPAN) Routing.

[15] Bormann C., Shelby Z., Chakrabarti S., Nordmark E. (2012). Neighbor Discovery Optimization for IPv6 over Low-Power Wireless Personal Area Networks (6LoWPANs).

[16] Ishaq I., Carels D., Teklemariam G.K., Hoebeke J., Abeele F.V.d., Poorter E.D., Moerman I., Demeester P. (2013). IETF standardization in the field of the internet of things (IoT): A survey. J. Sens. Actuator Netw..

[17] Ko J., Terzis A., Dawson-Haggerty S., Culler D.E., Hui J.W., Levis P. (2011). Connecting low-power and lossy networks to the internet. IEEE Commun. Mag..

[18] Watteyne T., Winter T., Barthel D., Dohler M. (2009). Routing Requirements for Urban Low-Power and Lossy Networks.

[19] Pister K., Phinney T., Thubert P., Dwars S. (2009). Industrial Routing Requirements in Low-Power and Lossy Networks.

[20] Porcu G., Buron J., Brandt A. (2010). Home Automation Routing Requirements in Low-Power and Lossy Networks.

[21] Martocci J., Mil P., Riou N., Vermeylen W. (2010). Building Automation Routing Requirements in Low-Power and Lossy Networks.

[22] Tavakoli A., Dawson-Haggerty S. (2009). Overview of Existing Routing Protocols for Low Power and Lossy Networks. Internet-Draft Draft-ietf-roll-protocols-survey-07.

[23] Sheng Z., Yang S., Yu Y., Vasilakos A., Mccann J., Leung K. (2013). A survey on the ietf protocol suite for the internet of things: Standards, challenges, and opportunities. IEEE Wirel. Commun..

[24] Alexander R., Brandt A., Vasseur J., Hui J., Pister K., Thubert P., Levis P., Struik R., Kelsey R., Winter T. (2012). RPL: IPv6 Routing Protocol for Low-Power and Lossy Networks.

[25] Iova O., Picco P., Istomin T., Kiraly C. (2016). RPL: The Routing Standard for the Internet of Things... Or Is It?. IEEE Commun. Mag..

[26] Oliveira L.M., De Sousa A.F., Rodrigues J.J. (2011). Routing and mobility approaches in IPv6 over LoWPAN mesh networks. Int. J. Commun. Syst..

[27] Kumar V., Tiwari S. (2012). Routing in IPv6 over low-power wireless personal area networks (6LoWPAN): A survey. J. Comput. Netw. Commun..

[28] Babu H.R., Dey U. (2014). Routing Protocols In IPv6 enabled LoWPAN: A Survey. Int. J. Sci. Res. Publ..

[29] Airehrour D., Gutierrez J., Ray S.K. (2016). Secure routing for internet of things: A survey. J. Netw. Comput. Appl..

[30] Borgia E. (2014). The Internet of Things vision: Key features, applications and open issues. Comput. Commun..

[31] Zanella A., Bui N., Castellani A., Vangelista L., Zorzi M. (2014). Internet of things for smart cities. IEEE Internet Things J..

[32] Sabbah A.I., El-Mougy A., Ibnkahla M. (2014). A survey of networking challenges and routing protocols in smart grids. IEEE Trans. Ind. Inform..

[33] Saputro N., Akkaya K., Uludag S. (2012). A survey of routing protocols for smart grid communications. Comput. Netw..

[34] Oliveira A., Vazão T. (2016). Low-power and lossy networks under mobility: A survey. Comput. Netw..

[35] Kim H.S., Ko J., Culler D.E., Paek J. (2017). Challenging the IPv6 Routing Protocol for Low-Power and Lossy Networks (RPL): A Survey. IEEE Commun. Surv. Tutor..

[36] Kamgueu P.O., Nataf E., Ndie T.D. (2018). Survey on RPL enhancements: A focus on topology, security and mobility. Comput. Commun..

[37] Das S.R., Perkins C.E., Belding-Royer E.M. (2003). Ad Hoc On-Demand Distance Vector (AODV) Routing.

[38] Alaa M., Zaidan A., Zaidan B., Talal M., Kiah M. (2017). A review of smart home applications based on Internet of Things. J. Netw. Comput. Appl..

[39] Zarpelão B.B., Miani R.S., Kawakani C.T., de Alvarenga S.C. (2017). A survey of intrusion detection in Internet of Things. J. Netw. Comput. Appl..

[40] Moy J. (1998). OSPF Version 2.

[41] Malkin G.S. (1998). RIP Version 2.

[42] Chakeres I., Perkins C. (2008). Dynamic MANET on-demand (DYMO) routing. Internet-Draft Draft-ietf-manet-dymo-15.

[43] Hu Y.C., Maltz D.A., Johnson D.B. (2007). The Dynamic Source Routing Protocol (DSR) for Mobile Ad Hoc Networks for IPv4.

[44] Kim K., Yoo S., Park J., Park S.D., Lee J. (2007). Hierarchical routing over 6LoWPAN (HiLow). Internet-Draft Draft-daniel-6lowpan-hilow-hierarchical-routing-01.txt.

[45] Kim K., Park S.D., Montenegro G., Yoo S., Kushalnagar N. (2007). 6LoWPAN Ad Hoc On-Demand Distance Vector Routing (LOAD). Internet-Draft Draft-daniel-6lowpan-load-adhoc-routing-03.txt.

[46] Kim K., Montenegro G., Park S., Chakeres I., Perkins C. (2007). Dynamic MANET On-demand for 6LoWPAN (DYMO-low) Routing. Internet-Draft Draft-montenegro-6lowpan-dymo-low-routing-03.txt.

[47] Tavakoli A., Dawson-Haggerty S., Hui J., Culler D. (2009). HYDRO: A hybrid routing protocol for lossy and low power networks. Internet-Draft Draft-tavakoli-hydro-01.txt.

[48] Dawson-Haggerty S., Tavakoli A., Culler D. Hydro: A hybrid routing protocol for low-power and lossy networks. Proceedings of the 2010 First IEEE International Conference on Smart Grid Communications (SmartGridComm).

[49] Iova O., Theoleyre F., Noel T. (2015). Using multiparent routing in RPL to increase the stability and the lifetime of the network. Ad Hoc Netw..

[50] Clausen T., Herberg U., Philipp M. A critical evaluation of the ipv6 routing protocol for low power and lossy networks (RPL). Proceedings of the 2011 IEEE 7th International Conference on Wireless and Mobile Computing, Networking and Communications (WiMob).

[51] Vasseur J., Agarwal N., Hui J., Shelby Z., Bertrand P., Chauvenet C. (2011). RPL: The IP Routing Protocol Designed for Low Power and Lossy Networks.

[52] Levis P., Clausen T.H., Gnawali O., Hui J., Ko J. (2011). The Trickle Algorithm.

[53] Mohamed B., Mohamed F. (2015). QoS Routing RPL for Low Power and Lossy Networks. Int. J. Distrib. Sens. Netw..

[54] Gara F., Ben Saad L., Ben Ayed R., Tourancheau B. RPL protocol adapted for healthcare and medical applications. Proceedings of the 2015 International Wireless Communications and Mobile Computing Conference (IWCMC).

[55] Thubert P. (2012). Objective Function Zero for the Routing Protocol for Low-Power and Lossy Networks (RPL).

[56] Gnawali O., Levis P. (2012). The Minimum Rank with Hysteresis Objective Function.

[57] Palattella M.R., Accettura N., Vilajosana X., Watteyne T., Grieco L.A., Boggia G., Dohler M. (2013). Standardized protocol stack for the internet of (important) things. IEEE Commun. Surv. Tutor..

[58] Barthel D., Vasseur J., Pister K., Kim M., Dejean N. (2012). Routing Metrics Used for Path Calculation in Low-Power and Lossy Networks.

[59] Clausen T., Yi J., Lavenu C., Lys A., Niktash A., Igarashi Y., Satoh H. (2011). The LLN On-demand Ad hoc Distance-vector Routing Protocol-Next Generation (LOADng). Internet-Draft Draft-Clausen-lln-Loadng-00.txt.

[60] Clausen T., Yi J., Niktash A., Igarashi Y., Satoh H., Herberg U., Lavenu C., Lys T., Dean J. (2016). The lightweight on-demand ad hoc distance-vector routing protocol-next generation (LOADng). Internet-Draft Draft-clausen-lln-loadng-15.txt.

[61] Goyal M., Baccelli E., Philipp M., Brandt A., Martocci J. (2013). Reactive Discovery of Point-to-Point Routes in Low-Power and Lossy Networks.

[62] Goyal M., Baccelli E., Brandt A., Martocci J. (2013). A Mechanism to Measure the Routing Metrics along a Point-to-Point Route in a Low-Power and Lossy Network.

[63] Barriquello C.H., Denardin G.W., Campos A. (2015). A geographic routing approach for IPv6 in large-scale low-power and lossy networks. Comput. Electr. Eng..

[64] Kuhn F., Wattenhofer R., Zollinger A. Worst-case optimal and average-case efficient geometric ad-hoc routing. Proceedings of the 4th ACM International Symposium on Mobile Ad Hoc Networking & Computing.

[65] Zhao M., Ho I.W.H., Chong P.H.J. (2016). An Energy-Efficient Region-Based RPL Routing Protocol for Low-Power and Lossy Networks. IEEE Internet Things J..

[66] Anamalamudi S., Zhang M., Sangi A.R., Perkins C.E., Anand S., (Remy) B.L. (2018). Asymmetric AODV-P2P-RPL in Low-Power and Lossy Networks (LLNs). Internet-Draft Draft-ietf-roll-aodv-rpl-03.

[67] Hui J., Kelsey R. (2016). Multicast Protocol for Low-Power and Lossy Networks (MPL).

[68] Oikonomou G., Phillips I., Tryfonas T. (2013). IPv6 Multicast Forwarding in RPL-Based Wireless Sensor Networks. Wirel. Pers. Commun..

[69] Dunkels A. (2011). The Contikimac Radio Duty Cycling Protocol.

[70] Dunkels A., Gronvall B., Voigt T. Contiki-a lightweight and flexible operating system for tiny networked sensors. Proceedings of the 29th Annual IEEE International Conference on Local Computer Networks.

[71] Abdel Fadeel K.Q., El Sayed K. ESMRF: Enhanced stateless multicast RPL forwarding for IPv6-based low-Power and lossy networks. Proceedings of the 2015 Workshop on IoT challenges in Mobile and Industrial Systems.

[72] Lorente G.G., Lemmens B., Carlier M., Braeken A., Steenhaut K. (2017). BMRF: Bidirectional Multicast RPL Forwarding. Ad Hoc Netw..

[73] Gaddour O., Koubâa A., Rangarajan R., Cheikhrouhou O., Tovar E., Abid M. Co-RPL: RPL routing for mobile low power wireless sensor networks using Corona mechanism. Proceedings of the 2014 9th IEEE International Symposium on Industrial Embedded Systems (SIES).

[74] Gaddour O., Koubâa A., Abid M. (2015). Quality-of-service aware routing for static and mobile IPv6-based low-power and lossy sensor networks using RPL. Ad Hoc Netw..

[75] Fotouhi H., Moreira D., Alves M. (2015). mRPL: Boosting mobility in the Internet of Things. Ad Hoc Netw..

[76] Fotouhi H., Zúñiga M., Alves M., Koubâa A., Marrón P. (2012). Smart-hop: A reliable handoff mechanism for mobile wireless sensor networks. Wireless Sensor Networks.

[77] Fotouhi H., Alves M., Zuniga Zamalloa M., Koubâa A. (2014). Reliable and fast hand-offs in low-power wireless networks. IEEE Trans. Mob. Comput..

[78] Bouaziz M., Rachedi A., Belghith A. (2019). EKF-MRPL: Advanced mobility support routing protocol for internet of mobile things: Movement prediction approach. Future Gener. Comput. Syst..

[79] Tahir Y., Yang S., McCann J. (2018). BRPL: Backpressure RPL for High-Throughput and Mobile IoTs. IEEE Trans. Mob. Comput..

[80] Tassiulas L., Ephremides A. (1992). Stability properties of constrained queueing systems and scheduling policies for maximum throughput in multihop radio networks. IEEE Trans. Autom. Control.

[81] Bouaziz M., Rachedi A., Belghith A., Berbineau M., Al-Ahmadi S. (2019). EMA-RPL: Energy and mobility aware routing for the Internet of Mobile Things. Future Gener. Comput. Syst..

[82] Yi J., Clausen T. (2014). Collection Tree Extension of Reactive Routing Protocol for Low-Power and Lossy Networks. Int. J. Distrib. Sens. Netw..

[83] Ko J., Jeong J., Park J., Jun J.A., Gnawali O., Paek J. (2015). DualMOP-RPL: Supporting Multiple Modes of Downward Routing in a Single RPL Network. ACM Trans. Sens. Netw. (TOSN).

[84] Kim H.S., Cho H., Kim H., Bahk S. (2017). DT-RPL: Diverse bidirectional traffic delivery through RPL routing protocol in low power and lossy networks. Comput. Netw..

[85] Taghizadeh S., Bobarshad H., Elbiaze H. (2018). CLRPL: Context-Aware and Load Balancing RPL for Iot Networks Under Heavy and Highly Dynamic Load. IEEE Access.

[86] Hou J., Jadhav R.A., Luo Z. (2017). Optimization of Parent-node Selection in RPL-based Networks. Internet-Draft Draft-hou-roll-rpl-parent-selection-00.

[87] Ancillotti E., Bruno R., Conti M. (2014). Reliable data delivery with the IETF routing protocol for low-power and lossy networks. IEEE Trans. Ind. Inform..

[88] Dorigo M., Birattari M. (2010). Ant Colony Optimization. Encyclopedia of Machine Learning.

[89] Qiu T., Lv Y., Xia F., Chen N., Wan J., Tolba A. (2016). ERGID: An efficient routing protocol for emergency response Internet of Things. J. Netw. Comput. Appl..

[90] Hossain A.K.M.M., Sreenan C.J., Alberola R.D.P. (2016). Neighbour-Disjoint Multipath for Low-Power and Lossy Networks. ACM Trans. Sens. Netw..

[91] Sobral J.V., Rodrigues J.J., Rabelo R.A., Filho J.C.L., Sousa N., Araujo H.S., Filho R.H. (2018). A framework for enhancing the performance of Internet of Things applications based on RFID and WSNs. J. Netw. Comput. Appl..

[92] EPCglobal (2008). EPCTM Radio-Frequency Identity Protocols Class-1 Generation-2 UHF RFID Protocol for Communications at 860 MHz–960 MHz Version 1.2.0.

[93] Intanagonwiwat C., Govindan R., Estrin D. Directed diffusion: A scalable and robust communication paradigm for sensor networks. Proceedings of the 6th Annual International Conference on Mobile Computing and Networking.

[94] Sasidharan D., Jacob L. (2018). Improving network lifetime and reliability for machine type communications based on LOADng routing protocol. Ad Hoc Netw..

[95] Sobral J.V.V., Rodrigues J.J.P.C., Rabelo R.A.L., Saleem K., Furtado V. (2019). LOADng-IoT: An Enhanced Routing Protocol for Internet of Things Applications over Low Power Networks. Sensors.

[96] Abreu C., Ricardo M., Mendes P. (2014). Energy-aware routing for biomedical wireless sensor networks. J. Netw. Comput. Appl..

[97] Capone S., Brama R., Accettura N., Striccoli D., Boggia G. An Energy Efficient and Reliable Composite Metric for RPL Organized Networks. Proceedings of the 2014 12th IEEE International Conference on Embedded and Ubiquitous Computing (EUC).

[98] Gaddour O., Koubaa A., Baccour N., Abid M. OF-FL: QoS-aware fuzzy logic objective function for the RPL routing protocol. Proceedings of the 2014 12th International Symposium on Modeling and Optimization in Mobile, Ad Hoc, and Wireless Networks (WiOpt).

[99] Kamgueu P.O., Nataf E., Ndie Djotio T. On design and deployment of fuzzy-based metric for routing in low-power and lossy networks. Proceedings of the 2015 IEEE 40th Local Computer Networks Conference Workshops (LCN Workshops).

[100] Araujo H.D.S., Filho R.H., Rodrigues J.J.P.C., Rabelo R.D.A.L., Sousa N.D.C., Filho J.C.C.L.S., Sobral J.V.V. (2018). A Proposal for IoT Dynamic Routes Selection Based on Contextual Information. Sensors.

[101] Chen Y., Chanet J.P., Hou K.M., Shi H., de Sousa G. (2015). A Scalable Context-Aware Objective Function (SCAOF) of Routing Protocol for Agricultural Low-Power and Lossy Networks (RPAL). Sensors.

[102] Gozuacik N., Oktug S. (2015). Parent-Aware Routing for IoT Networks. Internet of Things, Smart Spaces, and Next Generation Networks and Systems.

[103] Oliveira L., Rodrigues J.J.P.C., Kozlov S.A., Rabêlo R.A.L., Albuquerque V.H.C.d. (2019). MAC Layer Protocols for Internet of Things: A Survey. Future Internet.

[104] Hahm O., Baccelli E., Petersen H., Tsiftes N. (2016). Operating Systems for Low-End Devices in the Internet of Things: A Survey. IEEE Internet Things J..

[105] Musaddiq A., Zikria Y.B., Hahm O., Yu H., Bashir A.K., Kim S.W. (2018). A Survey on Resource Management in IoT Operating Systems. IEEE Access.

[106] Baccelli E., Hahm O., Gunes M., Wahlisch M., Schmidt T.C. RIOT OS: Towards an OS for the Internet of Things. Proceedings of the 2013 IEEE Conference on Computer Communications Workshops (INFOCOM WKSHPS).

[107] Antmicro RENODE. https://renode.io/.

